# Marginal structural models for repeated measures where intercept and slope are correlated: An application exploring the benefit of nutritional supplements on weight gain in HIV-infected children initiating antiretroviral therapy

**DOI:** 10.1371/journal.pone.0233877

**Published:** 2020-07-09

**Authors:** Ruth E. Farmer, Rhian Daniel, Deborah Ford, Adrian Cook, Victor Musiime, Mutsa Bwakura-Dangarembizi, Diana M. Gibb, Andrew J. Prendergast, A. Sarah Walker, on behalf of the ARROW trial team

**Affiliations:** 1 MRC Clinical Trials Unit at UCL, UCL Institute for Clinical Trials and Methodology, London, England, United Kingdom; 2 Department of Non Communicable Diseases Epidemiology, London School of Hygiene & Tropical Medicine, London, England, United Kingdom; 3 Division of Population Medicine, University of Cardiff, Cardiff, Wales, United Kingdom; 4 Joint Clinical Research Center, Kampala, Uganda; 5 Department of Paediatrics and Child Health, Makerere University College of Health Sciences, Kampala, Uganda; 6 University of Zimbabwe Clinical Research Centre, Harare, Zimbabwe; 7 Queen Mary University of London, London, England, United Kingdom; Albert Einstein College of Medicine, UNITED STATES

## Abstract

**Background:**

The impact of nutritional supplements on weight gain in HIV-infected children on antiretroviral treatment (ART) remains uncertain. Starting supplements depends upon current weight-for-age or other acute malnutrition indicators, producing time-dependent confounding. However, weight-for-age at ART initiation may affect subsequent weight gain, independent of supplement use. Implications for marginal structural models (MSMs) with inverse probability of treatment weights (IPTW) are unclear.

**Methods:**

In the ARROW trial, non-randomised supplement use and weight-for-age were recorded monthly from ART initiation. The effect of supplements on weight-for-age over the first year was estimated using generalised estimating equation MSMs with IPTW, both with and without interaction terms between baseline weight-for-age and time. Separately, data were simulated assuming no supplement effect, with use depending on current weight-for-age, and weight-for-age trajectory depending on baseline weight-for-age to investigate potential bias associated with different MSM specifications.

**Results:**

In simulations, despite correctly specifying IPTW, omitting an interaction in the MSM between baseline weight-for-age and time produced increasingly biased estimates as associations between baseline weight-for-age and subsequent weight trajectory increased. Estimates were unbiased when the interaction between baseline weight-for-age and time was included, even if the data were simulated with no such interaction. In ARROW, without an interaction the estimated effect was +0.09 (95%CI +0.02,+0.16) greater weight-for-age gain per month’s supplement use; this reduced to +0.03 (-0.04,+0.10) including the interaction.

**Discussion:**

This study highlights a specific situation in which MSM model misspecification can occur and impact the resulting estimate. Since an interaction in the MSM (outcome) model does not bias the estimate of effect if the interaction does not exist, it may be advisable to include such a term when fitting MSMs for repeated measures.

## Introduction

Ready-to-use therapeutic food, such as plumpy’nut (Nutriset, Rouen, France), is an energy-dense, peanut-based paste containing sugar, vegetable fat and skimmed milk powder, fortified with vitamins and minerals. It was designed, and is widely used, for nutritional rehabilitation of children with severe acute malnutrition. It is also widely used in stunted/wasted HIV-infected children entering antiretroviral therapy (ART) programmes [1], under the reasonable assumption that it will improve growth in children without severe acute malnutrition. Children rapidly gain weight after starting ART [2–5], but the causal role of nutritional supplements in this catch-up growth remains unclear.

**Fig 1:**
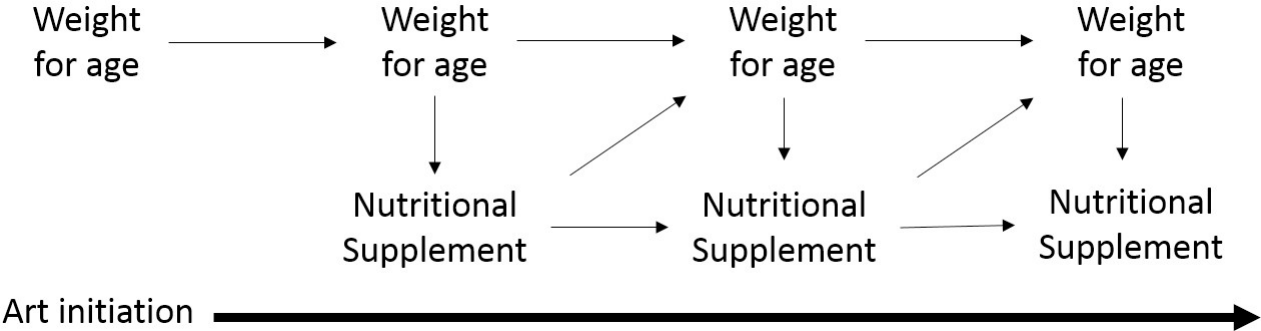
Graphical representation of assumed association between weight-for-age and use of nutritional supplementation following ART initiation in HIV-infected children.

ARROW was a randomised controlled trial investigating the impact of different management strategies in HIV-infected children starting ART in Uganda and Zimbabwe (ISCRTN 24791884) [1]. Within the protocol, plumpy’nut could be provided if considered clinically necessary based on the child’s weight-for-age and other measures of acute malnutrition; it was available through routine sources. As plumpy’nut was non-randomised and provided at varying times after ART initiation, evaluating the causal effect of supplementation on weight gain is complex. Weight-for-age is both the outcome of interest and a key time-dependent confounder (amongst others) of the association between cumulative supplement use and weight-for-age (Fig 1); that is, it is a risk factor for both the exposure and outcome of interest that changes through time. For example, CD4 count predicts both ART initiation and mortality, but also increases subsequent CD4 counts. In this situation, standard adjustments for time-varying CD4 will not correctly estimate the causal effect of ART on mortality, as part of the treatment effect mediated by CD4 will be removed. If there are further unmeasured common causes of CD4 and mortality, conditioning on CD4 will induce an association between exposure and these unmeasured common causes, introducing further bias [6–8]. In this application, weight-for-age is both the time-varying confounder and the outcome of interest.

Marginal structural models (MSMs) with inverse probability of treatment weighting (IPTW) are an established causal inference method to address time-dependent confounding [9], specifically IPTW of generalised estimating equations (GEEs) for repeated measures outcomes [10].

However, with repeated measures data, the underlying outcome trajectory (i.e. the trajectory in the absence of intervention) may depend on its starting value; that is, the intercept and slope may be correlated. For example, independent of supplement use, weight gain after ART initiation is likely accelerated in children who are initially very underweight due to their HIV infection, enabling them to “catch-up” at a faster rate than children only slightly underweight when they start ART.

Both the treatment model and the MSM must be correctly specified for unbiased causal inference from IPTW MSMs. Under randomisation, omitting a true correlation between intercept and slope from the MSM would not affect the treatment effect estimate. From the existing literature [10–12], the implications of such dependence between intercept and slope on correct estimation of the causal effect using IPTW MSM in observational repeated measures data are unclear, particularly since it is common to stabilise the IPTW with baseline covariates (which may include the baseline value of the outcome) to reduce the impact of extreme weights. This results in MSMs that in fact estimate a conditional treatment effect estimate. Although previous literature [10] highlights the importance of correct MSM specification, inclusion of interactions and baseline covariates in the MSM is discussed in relation to effect modification of the causal effect of interest only.

Here, we estimate the causal effect of nutritional supplementation on weight gain in HIV-infected children in their first year on ART, and investigate, using empirical observational data and simulation, the impact of incorrectly specifying correlated slope and intercept on estimated treatment effects from IPTW GEEs.

## Methods

ARROW methods and results have been reported previously [1]. In brief, children aged 3 months to 17 years, who met World Health Organization (WHO) 2006 criteria for initiating ART [13], were recruited following caregiver consent. Children were randomised to receive one of three combination ART regimens, and to routine laboratory versus clinically driven monitoring (factorial randomisation). During the first year on ART, no differences in any trial outcomes, including weight gain, were observed between randomised groups.

At enrolment (ART initiation) and at 4-weekly follow-up visits, data were collected on clinical and laboratory measures, including weight (from which a standardised score for weight-for-age is calculated), height and middle-upper-arm-circumference (MUAC). MUAC and calculated weight-for-height are both used to identify malnutrition. HIV-infected children are often both stunted and wasted so have more normal weight-for-height and therefore weight-for-age is also used when assessing malnutrition. Children considered to have malnutrition at any visit could be supplied with the protein-based supplement “plumpy’nut” at standard doses at the clinician’s discretion, provided this was available through routine sources (not provided by the trial). In Zimbabwe, children were referred to national malnutrition programmes for plumpy’nut. This analysis was therefore restricted to the three Ugandan study sites (Entebbe, Joint Clinical Research Centre (JCRC), Paediatric Infectious Diseases Clinic (PIDC)) that provided the supplement through the ARROW clinic and therefore recorded all use consistently. Analysis included only children enrolled after December 2007, when supplement use was added to case record forms. Finally, only children under 6 years at enrolment were included, as only 1/98 Ugandan children over 6 years were ever given plumpy’nut. Including older children would lead to large IPTW due to positivity assumption violation (essentially perfect prediction of treatment initiation) which may bias the causal estimate [9]. Follow-up was to the minimum of 12 months, death or withdrawal/lost to follow-up.

Data were organised into 4-weekly intervals, corresponding to scheduled visits. Few scheduled visits were missed (6%) and therefore missing values for covariates and plumpy’nut use were imputed by carrying the most recent observation forward when fitting the weighting models and calculating cumulative plumpy’nut use.

Due to positivity violations, it was not possible to model binary on/off treatment in each 4 weekly period. As such, weight estimation required the exposure process to be coarsened. The IPTW were calculated by first estimating the probability of initiating plumpy’nut in each monthly interval in those who had not yet initiated (“initiation model”), estimated using logistic regression. Baseline predictors of weight-for-age trajectory included in the initiation model were age, gender, CD4%, WHO stage, primary carer, weight-for-age (z-score), and a combined MUAC/weight-for-height indicator for malnutrition (< 0.8 times expected weight-for-height, or low MUAC (<12.5cm if <5 years, <14.5cm if 5–6 years); used when children were enrolled during 2007–2008). Time-dependent predictors were CD4%, previous weight-for-age, hospitalisation since last visit and MUAC/weight-for-height malnutrition indicator. Weight-for-age and weight-for-height z scores were calculated using WHO references [14]. CD4% and weight-for-age were included as linear continuous covariates. Time (weeks since ART initiation) was included as a linear spline with a knot at 16 weeks based on the trajectory of overall increases in weight-for-age [1, 5]. Age was included as a natural cubic spline with knots at the 25^th^, 50^th^ and 75^th^ percentiles. All baseline covariates and time were used to calculate numerators for the stabilised weights [10]. These weights were estimated separately by study site, to allow for potential differences in prescribing indications.

These initiation weights effectively assume a treatment probability of one for all visits after plumpy’nut is first started (i.e., that children remained on plumpy’nut continually post-initiation). As durations of plumpy’nut use varied (see S1 Table in S2 Appendix), we then estimated additional weights to account for stopping plumpy’nut (“stopping model”). Subsequent re-starting after stopping was only observed in 4 children and therefore was not considered further. Due to data sparsity, we could not model stopping at every visit after initiation or separately by site. 31% of children stopped plumpy’nut after just one month; therefore we calculated stopping weights based on the probability of continuing after 1 month’s use vs stopping after 1 month only. These probabilities were estimated from a logistic regression fitted for the month following plumpy’nut initiation only. Since this model was not compatible with modelling each child’s observed treatment duration, we assumed that children continuing had 6 months of plumpy’nut use in total, which was the median duration in those that did not stop after 1 month. To test the model’s sensitivity to this assumption, we also considered 3 and 9 months’ use in sensitivity analyses. Baseline predictors in the stopping model were: weight-for-age (continuous linear), centre, CD4% (<16%, 16–23%, >23%), primary carer, age (natural cubic spline), sex, WHO stage (4 vs lower), and MUAC/weight-for-height malnutrition indicator. Time-varying covariates included previous weight-for-age (continuous linear), change in CD4% from baseline (<0%, 0–15%,>15%), hospitalisation since last visit, and MUAC/weight-for-height malnutrition indicator. As for initiation weights, time was included as a linear spline with a knot at 16 weeks, and all baseline risk factors and time were used to calculate numerators for the stabilised weights [10]. Initiation and stopping weights were multiplied together to give the final IPTW. In those that never initiated plumpy’nut, their time-varying weight was determined by the initiation weight only. Loss-to-follow up/death was minimal (5% by one year), and therefore additional weighting to adjust for censoring was not included.

The effect of cumulative plumpy’nut use on change in weight-for-age (i.e. additional gain in weight-for-age per months’ extra plumpy’nut) was estimated using an IPTW GEE [10] with an independent working correlation matrix. We used an independent working correlation matrix in our analyses because exchangeable correlation structures in GEEs may give biased estimates when past outcome affects future exposure as is the case here [15, 16]. Other parametric associations with cumulative use (e.g. non-linear) were considered but there was no evidence that more complex relationships improved model fit (assessed via a wald test of all additional non-linear terms and examination of the magnitude of the estimates). All baseline covariates used for weight stabilisation were included in the GEE [17] and, as such, the estimand of interest was the average causal effect of 1 months plumpy’nut on weight-for-age conditional on all included baseline covariates listed above and time [11]. Weighted GEEs were compared to unweighted GEEs to assess the potential impact of time-dependent confounding on the estimated effect of supplement use on weight gain. Both weighted and unweighted GEEs were fitted with and without an interaction between time and baseline weight-for-age to allow for dependence between underlying weight gain (in the absence of supplementation) and baseline weight-for-age. Other interactions between baseline covariates and time or cumulative plumpy’nut use were included where heterogeneity p<0.05. Missed visits where weight-for-age had been carried forward (6% of observations) were not included in the GEEs.

The algebraic specification of all weights and the MSM are provided in S1 Appendix.

### Simulation study

Our focus was on estimating the treatment effect and, in particular, the bias associated with different analysis methods, rather than variance, which is important for ultimate inference but less so the greater the bias. We therefore simulated a single sample of 200,000 children, with normally distributed baseline weight-for-age z-scores (mean -2, variance 2.25). Simulation parameters for weight-for-age were chosen so that simulated data approximated ARROW data. Weight-for-age at monthly follow-up visits through 1 year was simulated to depend only on the previous weight-for-age and time, with no effect of supplement use, based on the observed mean change per month (0.1 z score increase per month). Supplement initiation was simulated to depend upon current weight-for-age and baseline weight-for-age. Stopping supplement was simulated to depend on current weight-for-age only (Eqs 1 & 2, Box 1). Simulation parameters for treatment initiation and subsequent stopping were not based on ARROW data, but chosen to produce time-dependent confounding without strong positivity violations. As a control simulation, supplement initiation was simulated to follow a sequential randomisation process with a fixed probability of initiation of 0.2 at each time-point for all who had not yet initiated (Eqs 3 & 4, Box 1) and all subjects remained on plumpy’nut for a minimum of 4 months or until 1 year from ART initiation.

The dependence between baseline weight-for-age and underlying weight gain (here equal to overall weight gain as data were simulated with no supplement effect) was then varied to assess its impact on the bias of the estimator of the causal effect of supplement use. This included a scenario where there was no dependence. This was done firstly by adding an interaction term into the underlying model, and secondly by drawing random intercepts (baseline) and slopes from a bivariate normal distribution (random slope distribution mean 0, and standard deviation (SD) based on the estimated SD of the slope in ARROW data). In sensitivity analyses, we repeated the simulations with varying effects of supplementation on weight gain.

Box 1 shows formal notation for the simulated data. Current weight-for-age was simulated as a function of previous and baseline weight-for-age, time, plus a random error. This ensured that current weight-for-age was associated with previous weight-for-age even after time and baseline weight-for-age were accounted for, as would occur in reality due to unmeasured factors (e.g. economic status, food security, family structures) that may also affect weight gain. All simulation code is provided in S4 Appendix.

Once the simulated data were obtained, we fitted the weighting models using the correct model specification, and then applied the estimated weights to the repeated measures MSM using generalised estimating equations as in the application. Two models were fitted to all simulation scenarios. In both, weight for age was the outcome, and plumpy’nut use, baseline weight for age, and time were fitted as main effects. An interaction between baseline weight for age and time was only included in the second model. As such, the estimator of interest in all simulation scenarios, including true randomisation of treatment, was the average treatment effect of plumpy’nut conditional on baseline weight-for-age and time.

**Box 1. Formal notation for simulated data.**

Main simulation: (1) Treatment allocation and (2) stopping dependent upon baseline and/or current weight-for-age. Control simulation: (3) Treatment allocated under sequential randomisation and stopping (4) dependent upon duration of use only

Define *W*_*ij*_ to be the random variable for weight-for age for subject *i* at time *j*(*j* = 0,1,2,…,12), with *W*_0_ therefore defined as the baseline weight-for age, and define *w*_*ij*_ to be the observed weight-for age for subject *i* at time *j*. Let *A*_*ij*_ be random variable representing treatment for subject *i* at time *j*, where *A* takes the value 0 or 1 to represent the presence of absence of nutritional supplements respectively. Then the data were simulated using parameters which reflected the data observed in the ARROW trial as follows, assuming an underlying weight-for-age increase of 0.1 units per month:

- Fixed effect model with interaction:
  $$W_{0}∼N(-2,\sigma^{2}=2.25)$$
  $$W_{ij}=w_{ij-1}+0.1+xw_{i0}+\gamma A_{ij-1}+\varepsilon_{ij}$$
  $$\varepsilon_{ij}∼N(0,0.09)\forall i,j$$
  *x* = 0, -0.05, -0.1, or -0.2 is the magnitude of the interaction between baseline weight and time, and *γ* = 0, 0.1, 0.2 or 0.3 is the treatment effect per month extra of supplement use.
- Random slope simulation model
  $$\begin{array}{c} W_{i0}\\ S_{i} \end{array}∼\left(\begin{array}{c} -2\\ 0 \end{array},\;(\begin{array}{cc} 2.25 & 0.06\rho \\ 0.06\rho & 0.0016 \end{array})\right)$$
  ρ=0,‐0.1,‐0.3,‐0.5,‐0.8or‐1,andγ=0,0.1,0.2or0.3
  $$W_{ij}=w_{ij-1}+(0.1+Si){+\gamma A_{ij-1}+\varepsilon }_{ij}$$
  $$\varepsilon_{ij}∼N(0,0.09)\forall i,j$$
  For both a) and b):
  $$logit\left(P\left(A_{ij}|A_{ij-1}=0\right)\right)=-1.5-1w_{ij}-0.1w_{i0}$$ (1)
  $$logit\left(P(A_{ij}=0|A_{ij-1}=1\right))=-1.38+0.5w_{ij}$$ (2)
  Or
  $$P\left(A_{ij}|A_{ij-1}=0\right)=0.2$$ (3)
  $$P(A_{ij}=0|A_{ij-1}=1)=0\;if\;\sum_{t=0}^{j}a_{ij-1}<4,\;\mathrm{and}\;1\;\mathrm{otherwise}$$ (4)

Four GEEs (with independent working correlation matrix) were then fitted to investigate biases in estimating the effect of plumpy’nut on weight gain.

Model-1: Unweighted GEE adjusting for visit and baseline weight-for-age

Model-2: Unweighted GEE adjusting for visit, baseline weight-for-age and their interaction

Model-3 IPTW (MSM) estimated via GEE, excluding an interaction term between baseline weight-for-age and visit in the MSM.

Model-4: IPTW (MSM) estimated via GEE including an interaction term between baseline weight-for-age and visit in the MSM.

All analyses were conducted using Stata version 14 [18].

The ARROW trial data used for this analysis are provided in S5 Appendix. Code to generate the simulated data are provided in S4 Appendix. This study is a re-analysis of data collected in a trial and so there is no specific ethical approval for the analysis. Caregivers provided written informed consent to enter the original trial. The original trial was approved by ethics committees in Uganda (Joint Clinical Research Centre Institutional Review Board), Zimbabwe (Medical Research Council of Zimbabwe), the UK (University College London Research Ethics Committee) and the US (Baylor College of Medicine Institutional Review Board).

## Results

### Cohort characteristics

**Fig 2:**
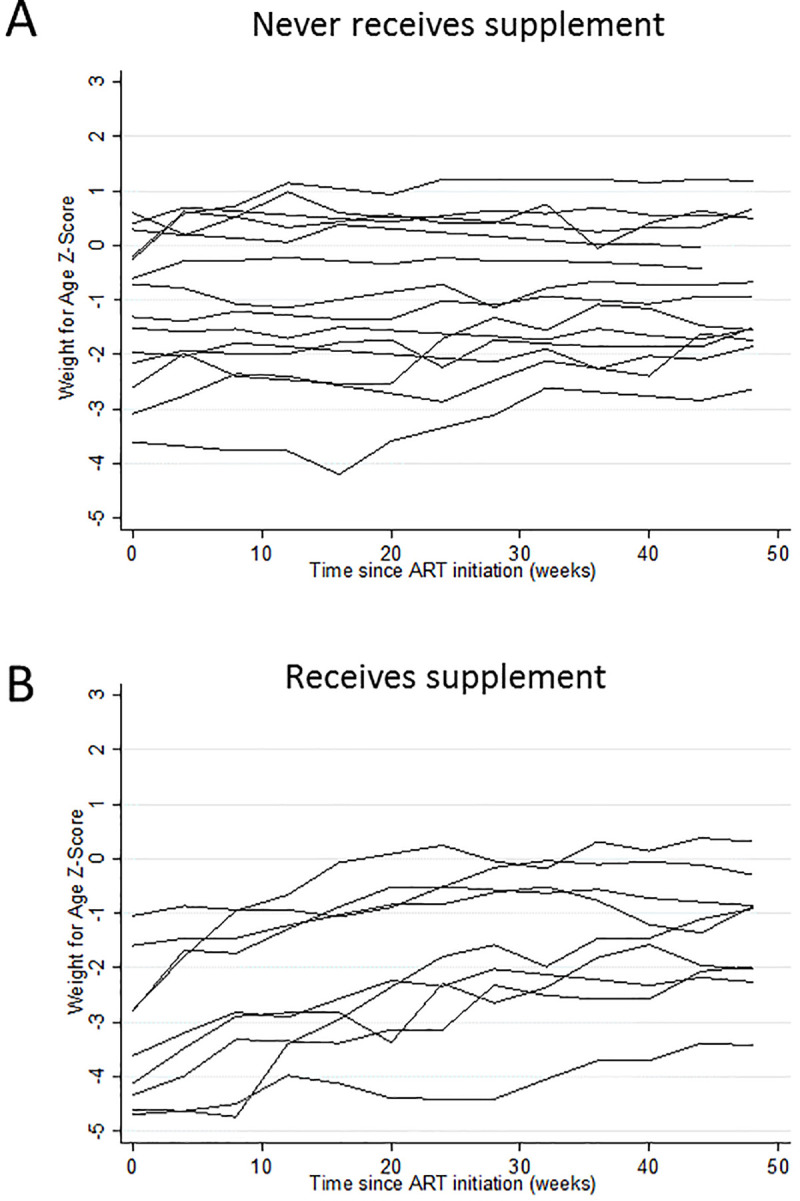
Random sample of trajectories of weight-for-age (Z-score) from children enrolled in ARROW trial after December 2007 from Ugandan sites (Entebbe, JCRC, PIDC) aged <6 years at enrolment, who never initiated plumpy’nut (A) and who initiated plumpy’nut at some point during the first year on ART (B).

385 Ugandan children were enrolled in ARROW during the study period. 98 were excluded as they were ≥6 years at enrolment, and 4 because they did not have baseline MUAC recorded. Of the 283 children included in the analysis, 63 (22%) initiated plumpy’nut before 12 months on ART, although one initiated it at the 12-month visit. As anticipated, children receiving plumpy’nut were younger, more malnourished and had more advanced HIV disease (Table 1). Fig 2 provides example trajectories of weight-for-age. Those ever prescribed plumpy’nut started and ended their first year on ART at lower weight-for-age, but also tended to have greater increases in weight-for-age. Excepting weeks 20 and 28, those initiating plumpy’nut at each monthly visit had a lower mean current and previous weight-for-age compared to non-initiators (Table 2). Although the single initiator at weeks 20 and 28 had a higher weight-for-age than the mean in non-initiators, their weight-for-age was reduced versus the previous month.

**Table 1:** Baseline (at ART initiation) characteristics of included children in the ARROW trial (enrolled after December 2007 aged <6 years at enrolment, from three study sites in Uganda (Entebbe, JCRC and PIDC)) (N = 284).

Mean (SD) [range] at ART initiation	Ever used plumpy’nut in first 48 weeks on ART		
	No (N = 221)	Yes (N = 63)	Total (N = 284)
**Age (years)**	2.89 (1.56)	1.86 (1.24)	2.66 (1.55)
	[0.4,5.92]	[0.49, 5.54]	[0.4, 5.92]
**Weight-for-age**	-1.58 (1.27)	-3.85 (1.38)	-2.08 (1.6)
	[-5.28, +2.29]	[-9.12, -1.03]	[-9.12, +2.29]
**Height-for-age**	-2.38 (1.45)	-3.62 (1.3)	-2.66 (1.51)
	[-7.39, +2.04]	[-7.07, -0.77]	[-7.39, +2.04]
**Weight-for-height**	-0.43 (1.29)	-2.59 (1.31)	-0.91 (1.57)
	[-5.36, +2.63]	[-4.75, +0.29]	[-5.36, +2.63]
**Middle upper arm circumference (MUAC) (cm)**	14.5 (2.3)	11.8 (1.7)	13.9 (2.4)
	[9.6, 36]	[8.2, 16]	[8.2, 36]
**CD4%^a^**	15.5 (8.2)	14.1 (8.0)	15.2 (8.1)
	[1, 42]	[1, 33]	[1, 42]
**N (%)**			
**Gender**			
** Male**	101 (46)	30 (48)	131 (46)
** Female**	120 (54)	33 (52)	153 (54)
**Primary Carer**			
** Mother**	158 (71)	48 (76)	206 (73)
** Other**	63 (29)	15 (24)	78 (27)
**WHO stage at ART initiation**			
** 1**	7 (3)	2 (3)	9 (3)
** 2**	98 (44)	4 (6)	102 (36)
** 3**	87 (39)	31 (49)	118 (42)
** 4**	29 (13)	26 (41)	55 (19)
**Low MUAC/weight-for-height composite^b^**			
** No**	181 (82)	14 (22)	195 (69)
** Yes**	40 (18)	48 (77)	88 (31)

^**a**^ truncated at 1^st^ and 99^th^ percentile.

^b^ composite score indicating presence of either low weight for height (< 0.8 x expected weight-for-height) or low MUAC for age (<12.5cm if <5 and <14.5cm if 5–6 years).

**Table 2:** Mean (SD) weight-for-age in initiators and non-initiators of plumpy’nut at each monthly visit in the ARROW trial (in those not yet initiated and not lost to follow up by this time).

	Current weight-for-age		Previous month’s weight-for-age	
Week since ART initiation	Non-initiator this month	Initiator this month	Non-initiator this month	Initiator this month
**0 (Baseline)**	n = 283	n = 0		
	-2.05 (1.52)			
**4**	n = 251	n = 32	n = 251	n = 32
	-1.69 (1.36)	-3.69 (1.12)	-1.82 (1.42)	-3.92 (0.98)
**8**	n = 236	n = 14	n = 236	n = 14
	-1.39 (1.22)	-3.36 (1.31)	-1.56 (1.27)	-3.68 (1.30)
**12**	n = 230	n = 4	n = 230	n = 4
	-1.24 (1.16)	-3.05 (0.69)	-1.35 (1.20)	-3.41 (1.20)
**16**	n = 227	n = 2	n = 227	n = 2
	-1.14 (1.11)	-2.45 (2.37)	-1.22 (1.14)	-3.22 (3.07)
**20**	n = 226	n = 1	n = 226	n = 1
	-1.09 (1.09)	-0.76	-1.14 (1.11)	-0.5
**24**	n = 225	n = 1	n = 225	n = 1
	-0.97 (1.07)	-2.78	-1.08 (1.09)	-1.89
**28**	n = 224	n = 1	n = 224	n = 1
	-0.95 (1.06)	-0.15	-0.98 (1.07)	0.17
**32**	n = 221	n = 3	n = 221	n = 3
	-0.88 (1.03)	-2.65 (1.70)	-0.92 (1.02)	-2.89 (2.20)
**36**	n = 221	n = 0	n = 221	n = 0
	-0.84 (0.98)		-0.88 (1.03)	
**40**	n = 218	n = 2	n = 218	n = 2
	-0.81 (0.95)	-1.99 (1.79)	-0.83 (0.98)	-1.99 (2.01)
**44**	n = 216	n = 2	n = 216	n = 2
	-0.77 (0.94)	-1.22 (0.37)	-0.81 (0.95)	-1.26 (0.29)
**48**	n = 209	n = 1	n = 209	n = 1
	-0.73 (0.88)	-2.74	-0.77 (0.91)	-4.14

### Application

Distributions of the stabilised initiation, stopping, and combined weights are shown in Table 3, with full weighting models in S2**–**S5 Tables in S2 Appendix. For the initiation weights for JCRC, baseline MUAC/weight-for-height indicator was a perfect predictor of receiving plumpy’nut and was therefore omitted from this weighting model. For each site, the mean initiation weight was close to 1, with none over 5 (Table 3). Similarly, the mean stopping and overall combined weight was approximately one. Since there were no extreme weights, they were not truncated.

**Table 3:** Distribution of stabilised IPTW estimated separately by study site in the ARROW trial.

	Mean	SD	Percentiles					Min	Max
**Weights for treatment initiation**			1^st^	10th	50th	90th	99th		
**Entebbe**	0.98	0.62	0.19	0.99	1.44	2.3	4.29	0.19	4.29
**JCRC**	0.99	0.33	0.26	1.00	1.04	1.24	2.19	0.26	3.83
**PIDC**	1.03	0.51	0.26	1.00	1.11	1.25	3.63	0.18	4.71
Weight for stopping after 1 month vs continue*									
**All centres combined**	1.02	0.43	0.34	0.78	0.92	1.00	1.01	0.34	3.67
**Combined start/stop weight**									
**All centres combined**	1.00	0.44	0.23	0.50	1.00	1.12	3.14	0.16	4.71

Factors included in each model are as follows: Denominator: baseline age, gender, baseline CD4%, baseline WHO stage, primary carer, baseline weight-for-age, and baseline MUAC/weight-for-height indicator for malnutrition (< 0.8 x expected weight-for-height or low MUAC (<12.5cm if <5 years or <14.5cm if 5–6 years)), CD4% at last visit, weight-for-age at last visit, hospitalisation since last visit and low MUAC/weight-for-height indicator at last visit. Numerator: baseline age, gender, baseline CD4%, baseline WHO stage, primary carer, baseline weight-for-age, and baseline MUAC/weight-for-height indicator for malnutrition (< 0.8 x expected weight-for-height or low MUAC (<12.5cm if <5 years or <14.5cm if 5–6 years)).

*(based on 62 data points of those who took plumpy’nut for 1 month or more before 12 months from starting ART.

Table 4 presents the estimated conditional average treatment effect of one month’s additional plumpy’nut use on a child’s expected change in weight-for-age per month for the four GEEs (full model outputs in S6 Table in S2 Appendix). Models 1 and 2 are estimated from an unweighted GEE, with independent working correlation matrix. Model 1 includes main effect adjustments for time since ART initiation, age, gender, baseline weight-for-age, baseline CD4%, baseline malnutrition indicator, baseline WHO stage, centre, and primary carer. Model 2 is as model 1 but also including an interaction between baseline weight-for-age and time in weeks since ART initiation. Models 3 and 4 are the IPTW equivalents of models 1 and 2 respectively. No other interaction terms tested had a heterogeneity of p<0.05 so were not included. In the weighted GEE without interactions between baseline weight-for-age and time (model 3), the estimates increase slightly compared to the unweighted model (model 1) although confidence intervals overlap, with an expected additional 0.09 (95% CI 0.02, 0.16) weight-for-age gain per month’s extra use of plumpy’nut. However, when baseline weight-for-age is allowed to independently affect weight-for-age trajectory through an interaction (model-4), the estimate of plumpy’nut effectiveness reduces to 0.03 (-0.04, +0.10), in fact very similar to the equivalent unweighted estimates (model 2). Without the interaction (model 3), the result of the IPTW GEE is borderline statistically significant; whilst no firm conclusions on a positive effect of plumpy’nut could be drawn, this is consistent with a previous study that found a positive effect of plumpy’nut using a different statistical approach [19]. However, in relative terms, as well as being far from statistically significant, the estimate of effect is reduced by around 67% when the interaction was included (model 4), leading to a qualitatively different interpretation. Sensitivity analyses assuming 3 or 9 rather than 6 months’ use in those not stopping after a one month gave similar results (S7 and S8 Tables in S2 Appendix)

**Table 4:** Estimates of effect of supplement use, time and baseline weight-for-age on growth per month from unweighted GEE (1,2) and IPTW weighted GEE (3,4) in the ARROW trial.

	Unweighted full baseline adjustment (1)	Unweighted full baseline adjustment with interaction (2)	IPTW weighted full baseline adjustment (3)	IPTW weighted full baseline adjustment with interaction (4)
	Estimate (95% Confidence Interval)	Estimate (95% Confidence Interval)	Estimate (95% Confidence Interval)	Estimate (95% Confidence Interval)
**Cumulative supplement use (per additional month of plumpy’nut)**	**0.065 (-0.002, 0.132)**	**0.022 (-0.047, 0.091)**	**0.086 (0.017, 0.155)**	**0.028 (-0.042, 0.098)**
**Time (per month up to 16 weeks, regardless of supplement use)**	0.041 (0.033, 0.048)	0.012 (0.002, 0.022)	0.040 (0.032, 0.048)	0.013 (0.003, 0.023)
**Time (per month post 16 weeks, regardless of supplement use)**	0.013 (0.010, 0.016)	-0.0004 (-0.004, 0.003)	0.014 (0.011, 0.017)	-0.001 (-0.005, 0.002)
**Baseline weight-for-age**	0.715 (0.644, 0.787)	0.990 (0.905, 1.075)	0.710 (0.636, 0.784)	0.984 (0.893, 1.074)
**Baseline weight-for-age time interaction (up to 16 weeks, regardless of supplement use)**		-0.015 (-0.020, -0.010)		-0.014 (-0.019, -0.009)
**Baseline weight-for-age time interaction (post 16 weeks, regardless of supplement use)**		-0.007 (-0.009, -0.005)		-0.008 (-0.010, -0.006)

(1) Adjusted for time, age at ART initiation, gender, baseline CD4%, baseline MUAC/weight-for-height indicator, baseline weight-for-age, primary carer and baseline WHO stage.

(2) (1) + interaction terms between baseline weight-for-age and both time terms.

(3) (1) including stabilised inverse probability of treatment weights.

(4) (2) including stabilised inverse probability of treatment weights.

All models assume that if plumpy’nut was not stopped after 1 month, it was continued for 6 months. For example, estimates from model (4) indicate that the impact of 1, 2, 3, 4, 5, 6 month’s plumpy’nut use is an additional +0.028, +0.056, +0.084, +0.112, +0.140, +0.168 unit’s weight-for-age Z-score respectively.

### Simulation study

**Fig 3:**
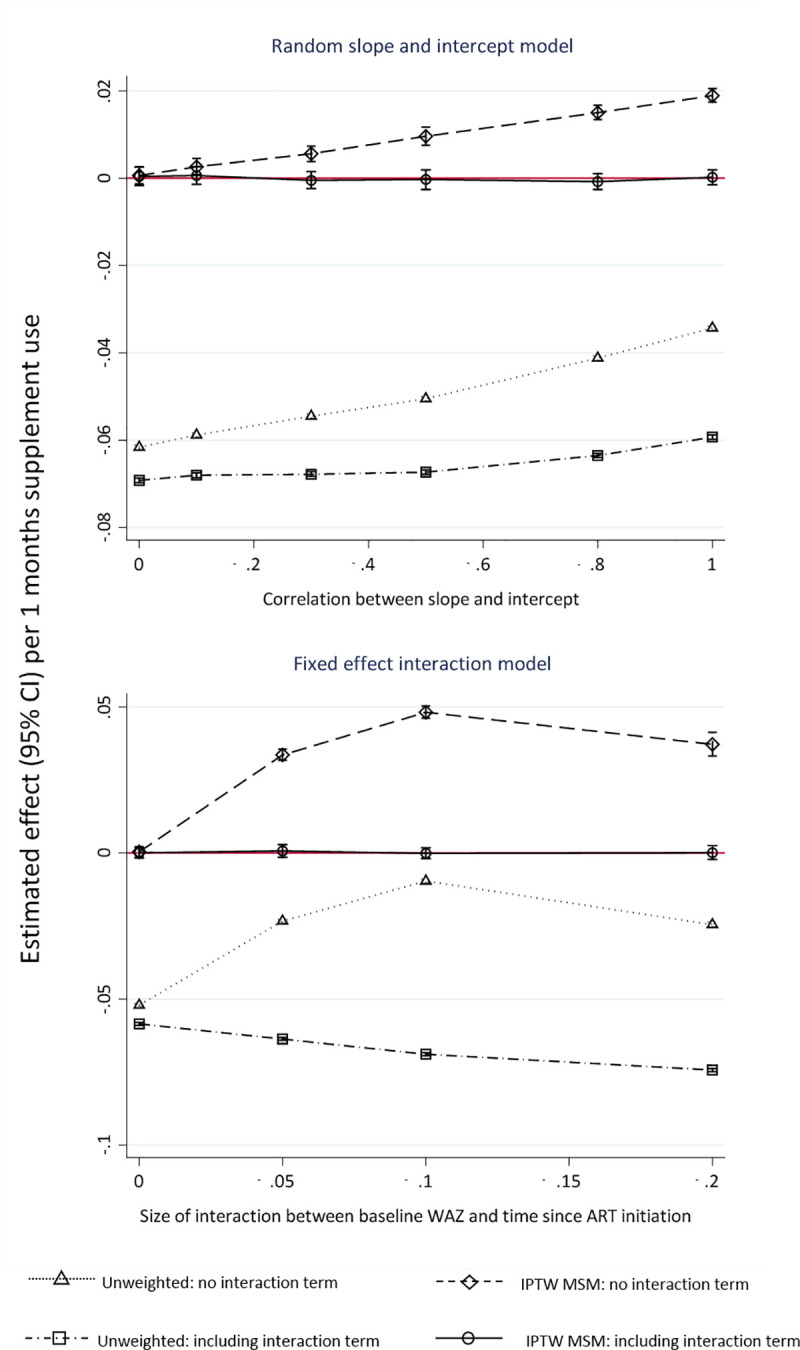
Effect (95% confidence intervals) of 1 month’s extra plumpy’nut on change in weight-for-age, where the true effect is zero, under different assumptions concerning the association between baseline weight-for-age and underlying weight-for-age trajectory (simulated data).

Simulation results were similar under both fixed and random effect simulation scenarios (Fig 3). Simulated weight-for-age trajectories were smoother than in ARROW as expected; and more simulated children ever received supplementation. For example in the random effect simulation, 60% and 95% of children received supplements in the first month or by one year respectively. These differences are likely because of the lack of other factors influencing treatment and weight gain in the relatively simple simulation, in contrast to the observed data.

Unweighted GEEs all showed very large negative bias, demonstrating the key impact of not adjusting for time-dependent confounding (Fig 3, S9 and S10 Tables in S3 Appendix). However, weighted MSM GEEs not including the interaction between baseline weight-for-age and time were positively biased, with bias increasing as the correlation between baseline weight-for-age and trajectory increased. In comparison, the weighted MSM GEEs including the interaction remained approximately unbiased (Fig 3, S9 and S10 Tables in S3 Appendix). The bias was greater in the simulation scenarios where the data were generated using an interaction term rather than a correlated random slope and intercept.

Association between baseline weight-for-age and slope simulated by a bivariate normal distribution (top) and a fixed effect interaction (bottom). Unweighted no interaction: GEE adjusting for time and baseline weight-for-age (analogous to Model 1 in Table 4). Unweighted with interaction: GEE adjusting for time, baseline weight-for-age and their interaction (Model 2 in Table 4). Weighted no interaction: MSM with IPTW excluding an interaction term between baseline weight-for-age and time in the MSM (Model 3 in Table 4). Weighted with interaction: MSM with IPTW including an interaction term between baseline weight weight-for-age and time in the MSM (Model 4 in Table 4). Red line indicates true simulated effect.]

The same pattern of results was observed when simulating a non-null treatment effect (S9 and S10 Tables in S3 Appendix). Simulating allocation of plumpy’nut randomly at each time point gave approximately unbiased estimates for all models, even those not including interaction terms (S1 Fig in S2 Appendix, S11 and S12 Tables in S3 Appendix).

## Discussion

This study aimed to estimate the causal effect of plumpy’nut on weight-for-age in HIV-infected children, because it remains uncertain whether nutritional supplements have any additional impact on weight gain in children starting ART. Lack of consistency of results from applying MSMs for repeated measures to this real-world example motivated an investigation of the potential impact of model misspecification due to correlated slope and intercept on bias in estimates from MSM with IPTW for repeated measures. Via a simple simulation, we showed that not incorporating correlation between slope and intercept via an interaction term in the MSM can result in biased causal effect estimates, and bias increases in magnitude with increasing strength of correlation. Further, under simulated randomisation, the same conditional average treatment effect estimate did not show bias even when the interaction was present in the data but omitted from the model. In ARROW, adding an interaction term to allow children with lower baseline weight-for-age to gain weight faster independently from supplement use, changed the conditional causal estimate from suggesting a small beneficial effect of supplement use, to no evidence of an effect of supplement use (actual estimate change from +0.09 to +0.03 per months’ extra use).

To our knowledge, this is the first study to specifically address the implications of dependence between intercept and slope in repeated measures data for correctly estimating causal effects in IPTW MSMs, and the first to estimate the effect of nutritional supplementation on weight gain in HIV-infected children using these causal methods. A previous study used a random effects model with random slope and intercept in a different dataset [19]. The authors did not specify whether they allowed for correlation between their random slope and intercept; however they estimated weight-for-age increased by 0.06 per additional months’ supplement use (95% CI 0.03,0.10). This is consistent with all estimates from the present study, but closest to the analyses that did not account for the correlation. However, this study included some older children aged 6–10 years at ART initiation, who were excluded from our analyses due to positivity violations. These estimates are therefore not directly comparable, although younger children would be expected to have received supplementation most commonly and therefore to drive any effects.

To further assess consistency with this previous study, we also fitted an unweighted mixed effects model, allowing random slopes and random intercepts. This suggested much larger beneficial effects of plumpy’nut on weight gain, with a 0.13 (95% CI 0.11,0.15) increase in weight-for-age per months’ additional plumpy’nut assuming independent intercept and slope, and 0.12 (0.10,0.14) allowing correlation between them. Although in the opposite direction of the expected time-dependent confounding in this context, these results are similar to those obtained from an unweighted GEE using an exchangeable working correlation matrix with no interaction in our study (0.14 (0.08,0.20)); which reduced to 0.06 (-0.01,+0.12) with interaction. We used an independent working correlation matrix in our analyses because exchangeable correlation structures in GEEs may give biased estimates when past outcome affects future exposure as here [15, 16]. It is possible that similar issues arise in the unweighted mixed effects model.

Both our simulation study and data analysis have limitations. Using a single large sample for the simulation study avoids small sample bias, but means we did not investigate precision or variance estimation/coverage. However, our main interest was investigating bias (since the main goal of IPTW is to reduce this), and the trends in the magnitude of the bias observed with increasing association between baseline and trajectory are still of interest even in the absence of information on precision. The simulation could have included other time-varying confounders (e.g. CD4%, hospitalisation) or baseline confounders (e.g. age, gender) of the association between plumpy’nut use and weight-for-age. This may have produced differences between models of more similar magnitude to those in our real-world application, though the bias in the simpler simulation is still demonstrated.

Strengths include high quality trial data with little missingness. Therefore, important potential confounders (both baseline and time-varying) were available to fit well-specified models for treatment initiation and outcome. However, data were insufficient to model treatment stopping flexibly, and we could only model continuing vs stopping after 1 month, and assume a fixed average duration of use in everyone that continued. Actual duration is likely informative in terms of weight-for-age gain, meaning some residual time-dependent confounding may remain. However sensitivity analyses assuming different durations of use gave similar conclusions clinically and statistically. The choice to impute missing visits in the weighting models using last observation carried forward was a pragmatic decision based on low numbers of missing visits and the already complex treatment process requiring the combination of two weighting models. Given that only 6% of visits were missed, it is unlikely that a substantial bias was introduced into the weights by using this approach. Use of additional weights for censoring or missingness may be useful to explore in further research as an alternative approach.

Other factors may also have been useful to further control for confounding in both initiation and stopping models, such as food insecurity, overall calorie intake or types of food available. Additionally, in one site, the baseline indicator for having low MUAC or height-for-age perfectly predicted treatment initiation, leading to a strict violation of the positivity assumption, and meaning this predictor could not be included in this initiation weighting model. However, it is unlikely that any serious residual time-dependent confounding remains due to its omission since, as a baseline variable, it was still included in the MSM. Lastly, also to avoid strict positivity violations, children aged 6 year or older at ART initiation were excluded from the analysis; our results are therefore generalizable only to children under 6 years.

The final MSM was considered to be the best possible parsimonious model, but it should be acknowledged that there may have been some remaining mis-specification, since tests for other interaction were based on Wald tests that may have been underpowered. Additionally, the difference between the standard and IPTW analyses in the empirical example were relatively small in magnitude. This could be because most children receiving the supplement initiated it in the first 3 months, and so the effect of post-baseline confounding was not as strong as initially hypothesised. The models with and without interactions between baseline weight-for-age and time produced what could be clinically relevant differences in the point estimates of the benefits of plumpy’nut, which are not explained by any of the limitations above. Due to some overlap in confidence intervals, random variation cannot be ruled out. However, the supporting simulation study identifies a clear problem from model misspecification when baseline and trajectories are associated and interactions are not included in the MSM.

For true causal interpretation, the underlying assumptions of conditional exchangeability, consistency, no interference and positivity are required to hold [20].Additionally, the correct model specification of both weighting and outcome models not only requires the correct terms in the model, but also that the terms are in the correct parametric form (e.g. linear vs quadratic). The limitations discussed above suggest that we cannot be confident of satisfying all of these assumptions, and so caution is needed in interpreting the estimate of the effect of plumpy’nut on weight gain as entirely causal. However, many of these limitations also apply to all other studies estimating effects from multivariable models. Notwithstanding, this application is still a useful example of how results may change if the MSM is incorrectly specified in this way. Here, the overall conclusion is only slightly altered (borderline to no evidence of a beneficial effect), and the bias was quite small in magnitude. However, in other situations the bias could substantially alter conclusions, and there are many other contexts where our methodological findings may have implications. For example, in untreated asthma, forced expiratory volume (FEV) is much lower in severe asthma than mild asthma. The impact of standard asthma medication (analogous to ART) may have a greater impact on improving severe asthma than mild asthma, and therefore the slope of change in FEV will be steeper for those starting at a lower value. Any study using MSMs to investigate the impact of concomitant medications on FEV change, in addition to standard therapy, would therefore need to consider this.

Our findings suggest that HIV-infected children initiating ART with low weight-for-age have no additional weight gain due to nutritional supplementation. This contrasts prior studies suggesting benefits of nutritional supplementation but without adjusting for time-varying confounders [19]. Our data suggest that growth reconstitution in HIV-infected children is mediated primarily by ART. Weight loss in untreated, HIV-infected children is driven by several factors, including anorexia, reduced intake due to oral pathology, malabsorption, cytokine-mediated muscle loss, increased metabolic demands and opportunistic infections [21]. ART initiation leads to reduced immune activation, improved appetite, intestinal repair, and resolution of opportunistic infections. It appears that the additional nutrition provided by supplementary food does not significantly improve weight gain at a time when ART is fundamentally altering the physiological milieu, enabling growth reconstitution.

In summary, correct specification of both the IPTW and MSM models is a necessary assumption for causal interpretation of the resulting estimate. This study highlights a specific situation in which MSM model misspecification may occur and have an impact on the resulting estimate. Due to such misspecification being irrelevant under true randomisation, the importance of accounting for correlated slope and intercept may not be immediately obvious to those using MSMs for repeated measures. Additional simulation studies that assess more complex model specifications, variance, mean square error and coverage are required to provide concrete recommendations, but our initial findings suggest that since an interaction in the MSM (outcome) model does not appear to bias the estimate of effect if the interaction does not exist, it may be advisable to include such a term when fitting MSMs for repeated measures.

## Supporting information

S1 Appendix Algebraic specification of weights and MSMs. (DOCX)

S2 Appendix S1–S8 Tables and S1 Fig. (DOCX)

S3 Appendix S9–S12 Tables. (XLSX)

S4 Appendix Simulation code. (DOCX)

S5 Appendix ARROW trial data. (XLSX)

S6 Appendix ARROW trial team. (DOCX)
