# Supplementary material for: Marginal structural models for repeated measures where intercept and slope are correlated: An application exploring the benefit of nutritional supplements on weight gain in HIV-infected children initiating antiretroviral therapy
Source: PLoS One. 2020 Jul 9;15(7):e0233877. doi: 10.1371/journal.pone.0233877 (PMC7347189; doi:10.1371/journal.pone.0233877)
Supplement: S1 Appendix — (DOCX) [file pone.0233877.s001.docx]

**Algebraic specification of the weights**

Let $T$ be the random variable (r.v) for the weekly interval after ART initiation, where the observed week $t$ takes the values $1 (0-4 weeks), 2 (4-8 weeks)\ldots12 (44-48 weeks)$. In the observed data, no children were initiated on plumpy’nut at the time of ART initiation.

Let A be the (r.v) representing initiation of plumpy’nut, where $A(t)$ represents whether a child has initiated by time $T=t$, taking the observed values $a(t)=0 or 1$. Therefore once $a(t)=1,$ $a(t)=1 for all T>t.$

Let $S$ be the r.v denoting whether a child is on or off plumpy’nut at each interval following initiation. S(t) can take the observed values$s(t)=0$(off) or $s(t)=1$ (on).

We further denote $Cum\left( A(t) \right)$ to be the cumulative amount of plumpy’nut given up to and including time $t$. For clarity, this implies that if plumpy’nut is initiated at time $t$, then $Cum\left( A(t) \right)=1$.

As outlined in the main text, we assume that a child can either stop plumpy’nut after 4 weeks, or continue for 24 weeks (or until end of their 1 year follow up if this is less).

Specifically:

If $S\left( t \right)=0$when $Cum\left( A\left( t-1 \right)=1 \right)$, then $S\left( t \right)=0$ for all $T>t$ and $Cum\left( A\left( t \right)=1 \right)$ for all$T>t-1$.

If $S\left( t \right)=1$when $Cum\left( A\left( t-1 \right)=1 \right)$, then $Cum\left( A(t) \right)$increases by 1 for each interval $T>t-1$, until reaching a maximum of 6, and $S\left( t \right)=1$ for$t+1, t+2, t+3, t+4$, and 0 thereafter.

Let $V$ be r.v. for the vector of baseline covariates (which includes baseline weight-for-age) and $L$ be the vector of time dependent covariates (which includes weight-for-age and time), where $L(t)$ is the vector of values at time $t$and $\underline{L}(t)$ represents the full time dependent covariate history to time $t$.

Finally, we let the study site be denoted by $J$, taking the observed values $j = 1, 2, 3$

We calculate a child’s weight for **starting** plumpy’nut in those who are yet to initiate (i.e. we set $Cum\left( A(0) \right)=0$ and compute for all intervals where $cum\left( A\left( t-1 \right) \right)=0)$as follows:

$${W\left( t \right)}_{j}^{A}= \prod_{k=1}^{t} \frac{P\left[ Cum\left( A\left( t-1 \right) \right)=0,V,J=j \right]}{P\left[ Cum\left( A\left( t-1 \right) \right)=0,\underline{L}\left( k-1 \right),V, J=j \right]}$$

Post initiation, we apply the following:

$${W\left( t \right)}_{j}^{A}= {W\left( t-1 \right)}_{j}^{A}$$

We calculate a child’s weight for **stopping** plumpy’nut in the interval following initiation (when $Cum\left( A\left( t-1 \right)=1 \right)$ and $A\left( t-2 \right)=0)$only (i.e. stopping after 4 weeks versus continuing), as follows:

${W(t)}^{S}=\frac{P [S\left( t \right)|Cum(A\left( t-1 \right)=1),A(t-2)=0,V]}{P[S\left( t \right)|Cum\left( A\left( t-1 \right)=1 \right),A(t-2)=0,\underline{L}\left( t-1 \right)]}$

We set ${W\left( 1 \right)}^{S}=1$

Prior to and during the interval of plumpy’nut initiation (i.e. when$cum(A\left( t-1 \right))=0$) we also set ${W\left( t \right)}^{S}=1$.

If initiation is observed in interval $T=k$, then for all intervals $T>k+1$, we set ${W(t)}^{S} = {W(t-1)}^{S}$.

In other words, the probabilities for the weights for stopping plumpy’nut are only estimated in the first 4-week interval after plumpy’nut was initiated. For later intervals, $S(t)$ is fixed and therefore the weight remains constant. A child that never initiates plumpy’nut would have a constant weight ${W(t)}^{S}=1$ for all intervals.

We then calculate the **overall inverse probability weight** for a child at time t to be ${W(t)}_{j}^{A}{\times W(t)}^{S}$

**Algebraic specification of the MSM**

Denoting $X$ to be the r.v. representing baseline weight for age (taking the realisation $X=x$), and $V$ to be the vector of baseline covariates used in the weighting models (that also includes baseline weight for age), we then fit the following two MSMs to model weight-for-age (represented by the r.v $Y$) at time $t$:

1. No interaction included:

$E\left[ Y | V , t \right]= \alpha+ \beta Cum(A\left( t-1 \right))+ \gamma t+ \zeta V$

1. Interaction included:

$E\left[ Y | V, t \right]= \alpha+ \beta Cum\left( A\left( t-1 \right) \right)+ \gamma t+ \zeta V+\delta xt$

In both models, the target parameter is: the conditional average treatment effect per month increase in plumpy’nut use.

Because the MSM at time $t$ depends on $Cum(A(t-1)),$ the weight applied in the MSM at time $t$is ${W(t-1)}_{j}^{A}{W(t-1)}^{S}$
