# Supplementary material for: Marginal structural models for repeated measures where intercept and slope are correlated: An application exploring the benefit of nutritional supplements on weight gain in HIV-infected children initiating antiretroviral therapy
Source: PLoS One. 2020 Jul 9;15(7):e0233877. doi: 10.1371/journal.pone.0233877 (PMC7347189; doi:10.1371/journal.pone.0233877)
Supplement: S2 Appendix — (DOCX) [file pone.0233877.s002.docx]

| **Total months of plumpy’nut use during first 12 months on ART in those that initiated** | **Entebbe** | **JCRC** | **PIDC** | **Total** | **Cumulative total** | **Cumulative %** |
| --- | --- | --- | --- | --- | --- | --- |
| **1** | 3 | 2 | 14 | 19 | 19 | 31% |
| **2** | 1 | 0 | 1 | 2 | 21 | 34% |
| **3** | 0 | 3 | 2 | 5 | 26 | 42% |
| **4** | 4 | 2 | 3 | 9 | 35 | 56% |
| **5** | 2 | 1 | 2 | 5 | 40 | 64% |
| **6** | 1 | 1 | 1 | 3 | 43 | 69% |
| **7** | 1 | 2 | 1 | 4 | 47 | 76% |
| **8** | 0 | 1 | 0 | 1 | 48 | 77% |
| **9** | 2 | 0 | 1 | 3 | 51 | 82% |
| **10** | 2 | 0 | 0 | 2 | 53 | 85% |
| **11** | 9 | 0 | 0 | 9 | 62 | 100% |

**Table S1:** Total duration of plumpy’nut use (in months) in the first 12 months on ART, for those who start plumpy’nut at all.

|  | Denominator Model | | | Numerator Model | | |
| --- | --- | --- | --- | --- | --- | --- |
|  | OR | SE | 95% CI | OR | SE | 95% CI |
| Time (per month up to 16 weeks) | 0.74 | 0.09 | (0.57 , 0.95) | 1.02 | 0.05 | (0.93 , 1.13) |
| Time (per month post 16 weeks) | 0.97 | 0.08 | (0.83 , 1.14) | 0.88 | 0.06 | (0.78 , 1.01) |
| Weight-for-age z score at previous visit (per unit change) | 0.66 | 0.57 | (0.12 , 3.59) |  |  |  |
| CD4 (%) at previous visit (per %) | 1.06 | 0.11 | (0.87 , 1.29) |  |  |  |
| Hospitalisation since last visit (Yes vs No) | 10.33 | 20.37 | (0.22 , 493.33) |  |  |  |
| Joint MUAC/weight-for-height indicator for malnutrition at previous visit | 0.35 | 0.54 | (0.02 , 7.13) |  |  |  |
| Primary carer (mother vs other) | 1.16 | 0.89 | (0.26 , 5.25) | 0.94 | 0.59 | (0.27 , 3.24) |
| Baseline WHO Stage (4 vs 1,2 or 3) | 1.69 | 1.61 | (0.26 , 10.95) | 0.84 | 0.59 | (0.21 , 3.32) |
| Baseline Age (spline term 1) | 0.66 | 0.60 | (0.11 , 3.95) | 0.98 | 0.70 | (0.24 , 4.01) |
| Baseline age (spline term 2) | 1.03 | 0.21 | (0.69 , 1.54) | 0.97 | 0.16 | (0.7 , 1.33) |
| Gender (F v M) | 4.64 | 3.34 | (1.13 , 19.02) | 3.58 | 2.02 | (1.18 , 10.85) |
| Baseline Joint MUAC/weight-for-height indicator for malnutrition | 2.61 | 4.38 | (0.10 , 69.73) | 0.42 | 0.14 | (0.22 , 0.80) |
| Baseline CD4 (%) (per %) | 0.87 | 0.10 | (0.69 , 1.09) | 2.25 | 1.99 | (0.40 , 12.67) |
| Baseline weight-for-age z score (per unit change) | 0.48 | 0.44 | (0.08 , 2.89) | 0.94 | 0.03 | (0.87 , 1.01) |
| Constant term | 0.14 | 0.33 | (0.00 , 14.25) | 0.01 | 0.02 | (0.00 , 0.35) |

**Table S2: Models for stabilised IPTW for plumpy’nut initiation in Entebbe**

Baseline age entered as a natural cubic spline with knots at 25^th^, 50^th^ and 75^th^ percentiles.

MUAC/weight-for-height indicator for malnutrition (< 0.8 x expected weight-for-height or low MUAC (<12.5cm if <5 years or <14.5cm if 5-6 years))

|  | Denominator Model | | | Numerator Model | | |
| --- | --- | --- | --- | --- | --- | --- |
|  | OR | SE | 95% CI | OR | SE | 95% CI |
| Time (per month up to 16 weeks) | 0.65 | 0.54 | (0.13 , 3.27) | 0.99 | 0.06 | (0.88 , 1.12) |
| Time (per month post 16 weeks) | 0.83 | 0.10 | (0.65 , 1.06) | 0.83 | 0.11 | (0.65 , 1.07) |
| Weight-for-age at previous visit z score (per unit change) | 1.01 | 0.11 | (0.81 , 1.25) |  |  |  |
| CD4 (%) at previous visit (per % ) | 3.75 | 4.29 | (0.4 , 35.25) |  |  |  |
| Hospitalisation since last visit (Yes vs No) | 1.26 | 1.65 | (0.1 , 16.57) |  |  |  |
| Joint MUAC/weight-for-height indicator for malnutrition at previous visit | 0.86 | 0.11 | (0.66 , 1.11) |  |  |  |
| Primary carer (mother vs other) | 1.62 | 1.87 | (0.17 , 15.48) | 1.66 | 1.74 | (0.21 , 12.97) |
| Baseline WHO Stage (4 vs 1&2) | 1.19 | 1.30 | (0.14 , 10.04) | 1.69 | 1.71 | (0.23 , 12.28) |
| Baseline WHO Stage (3 vs 1&2) | 1.32 | 1.55 | (0.13 , 13.17) | 1.87 | 2.02 | (0.23 , 15.46) |
| Baseline Age (spline term 1) | 1.61 | 1.47 | (0.27 , 9.61) | 1.43 | 1.08 | (0.33 , 6.26) |
| Baseline age (spline term 2) | 0.77 | 0.20 | (0.46 , 1.29) | 0.81 | 0.18 | (0.52 , 1.25) |
| Gender (F v M) | 0.52 | 0.45 | (0.09 , 2.86) | 0.59 | 0.47 | (0.12 , 2.81) |
| Baseline Joint MUAC/weight-for-height indicator for malnutrition | omitted |  |  | omitted |  |  |
| Baseline CD4 (%) (per %) | 1.21 | 0.15 | (0.94 , 1.55) | 1.06 | 0.07 | (0.93 , 1.2) |
| Baseline weight-for-age Z score (per unit change) | 0.38 | 0.30 | (0.08 , 1.75) | 0.28 | 0.10 | (0.14 , 0.56) |
| Constant term | 0.001 | 0.0035 | (0 , 1.0339) | 0.0002 | 0.0006 | (0 , 0.068) |

**Table S3: Models for stabilised IPTW for plumpy’nut initiation in JCRC**

Baseline age entered as a natural cubic spline with knots at 25^th^, 50^th^ and 75^th^ percentiles.

MUAC/weight-for-height indicator for malnutrition (< 0.8 x expected weight-for-height or low MUAC (<12.5cm if <5 years or <14.5cm if 5-6 years)); at baseline this factor was a perfect predictor of initiation and was therefore not included

|  | Denominator Model | | | Numerator Model | | |
| --- | --- | --- | --- | --- | --- | --- |
|  | OR | SE | 95% CI | OR | SE | 95% CI |
| Time (per month up to 16 weeks) | 0.54 | 0.18 | (0.29 , 1.02) | 1.01 | 0.04 | (0.93 , 1.1) |
| Time (per month post 16 weeks) | 0.99 | 0.08 | (0.85 , 1.15) | 0.98 | 0.03 | (0.94 , 1.04) |
| Weight-for-age at previous visit z score (per unit change) | 1.01 | 0.03 | (0.95 , 1.08) |  |  |  |
| CD4 (%) at previous visit (per % ) | 1.04 | 0.04 | (0.97 , 1.12) |  |  |  |
| Hospitalisation since last visit (Yes vs No) | 1.17 | 0.81 | (0.3 , 4.56) |  |  |  |
| Joint MUAC/weight-for-height indicator for malnutrition at previous visit | 3.83 | 2.82 | (0.9 , 16.25) |  |  |  |
| Primary carer (mother vs other) | 0.50 | 0.40 | (0.1 , 2.38) | 0.61 | 0.42 | (0.16 , 2.34) |
| Baseline WHO Stage (4 vs 1&2) | 2.54 | 1.72 | (0.68 , 9.56) | 1.71 | 1.12 | (0.48 , 6.14) |
| Baseline WHO Stage (3 vs 1&2) | 0.77 | 0.58 | (0.18 , 3.36) | 0.76 | 0.55 | (0.18 , 3.16) |
| Baseline Age (spline term 1) | 1.02 | 0.50 | (0.39 , 2.66) | 0.91 | 0.40 | (0.38 , 2.16) |
| Baseline age (spline term 2) | 0.75 | 0.14 | (0.52 , 1.1) | 0.80 | 0.14 | (0.56 , 1.13) |
| Gender (F v M) | 1.06 | 0.55 | (0.38 , 2.95) | 1.26 | 0.59 | (0.5 , 3.15) |
| Baseline Joint MUAC/weight-for-height indicator for malnutrition | 1.56 | 1.17 | (0.36 , 6.82) | 2.40 | 1.70 | (0.6 , 9.66) |
| Baseline CD4 (%) (per %) | 1.00 | 0.04 | (0.92 , 1.09) | 1.04 | 0.03 | (0.98 , 1.1) |
| Baseline weight-for-age Z score (per unit change) | 0.72 | 0.25 | (0.37 , 1.43) | 0.44 | 0.11 | (0.27 , 0.74) |
| Constant term | 0.0009 | 0.0016 | (0 , 0.0341) | 0.0014 | 0.0022 | (0.0001 , 0.0297) |

**Table S4: Models for stabilised IPTW for plumpy’nut initiation in PIDC**

Baseline age entered as a natural cubic spline with knots at 25^th^, 50^th^ and 75^th^ percentiles.

MUAC/weight-for-height indicator for malnutrition (< 0.8 x expected weight-for-height or low MUAC (<12.5cm if <5 years or <14.5cm if 5-6 years))

|  | **Denominator Model** | | | **Numerator Model** | | |
| --- | --- | --- | --- | --- | --- | --- |
|  | OR | SE | 95% CI | OR | SE | 95% CI |
| **Week (up to 16 weeks)** | 0.85 | 0.21 | [0.52 , 1.37] | 0.95 | 0.17 | [0.67 , 1.35] |
| **Week (16 weeks onwards)** | 0.81 | 0.13 | [0.6 , 1.11] | 0.79 | 0.12 | [0.58 , 1.07] |
| **Centre (vs entebbe)** |  |  |  |  |  |  |
| JCRC | 0.52 | 0.96 | [0.01 , 19.58] | 0.31 | 0.47 | [0.02 , 6.06] |
| PIDC | 0.06 | 0.09 | [0 , 1.55] | 0.06 | 0.09 | [0 , 1.06] |
| **Weight for age at previous visit (per unit change in Z score)** | 0.53 | 0.57 | [0.07 , 4.3] |  |  |  |
| **Hospital visit since previous visit (Y v N)** | 0.52 | 0.89 | [0.02 , 14.79] |  |  |  |
| **Malnourished at previous visit (Y V N)** | 2.34 | 4.5 | [0.06 , 97.32] |  |  |  |
| **Baseline WHO stage (4 vs 1,2,3)** | 0.06 | 0.09 | [0 , 0.98] | 0.08 | 0.10 | [0.01 , 0.95] |
| **Primary carer (Mother vs other)** | 0.74 | 1.03 | [0.05 , 11.37] | 0.79 | 0.80 | [0.11 , 5.77] |
| **Baseline age (linear)** | 0.16 | 0.24 | [0.01 , 3.04] | 0.12 | 0.14 | [0.01 , 1.31] |
| **Baseline age (additional spline term)** | 1.4 | 0.52 | [0.68 , 2.9] | 1.47 | 0.45 | [0.8 , 2.67] |
| **Change in CD4 % from baseline (vs any decrease)** |  |  |  |  |  |  |
| 0-15% increase | 11.58 | 20.2 | [0.38 , 354.87] |  |  |  |
| >15% increase | 2.04 | 5.4 | [0.01 , 371.63] |  |  |  |
|  |  |  |  |  |  |  |
| **Baseline CD4 % (vs <16)** |  |  |  |  |  |  |
| 16-23 | 31.73 | 95.4 | [0.09 , 11531.63] | 40.32 | 121.0 | [0.11 , 14427.56] |
| >23 | 2.26 | 3.9 | [0.08 , 66.78] | 1.28 | 1.8 | [0.08 , 20.96] |
| **Sex (FvM)** | 1.18 | 1.3 | [0.13 , 10.46] | 1.24 | 1.2 | [0.19 , 7.95] |
| **Baseline Weight for age (per unit change in z score)** | 0.89 | 0.84 | [0.14 , 5.69] | 0.43 | 0.26 | [0.14 , 1.38] |
| **Constant** | 237.8 | 1048.5 | [0.04 , 1345526] | 127.88 | 424.1 | [0.19 , 84992.36] |

**Table S5: Models for stabilised IPTW for continuing plumpy’nut after 1 month vs stopping.** Model fitted in 62 children at the visit after first recieving plumpy’nut. Weight of 1 assigned to all intervals prior to and including the first month of plumpy’nut initiation.

Baseline age entered as a natural cubic spline with knots at 25^th^, 50^th^ and 75^th^ percentiles.

MUAC/weight-for-height indicator for malnutrition (< 0.8 x expected weight-for-height or low MUAC (<12.5cm if <5 years or <14.5cm if 5-6 years))

|  | **Un-weighted** | | **IPTW** | |
| --- | --- | --- | --- | --- |
|  | **No interaction** | **Interaction** | **No interaction** | **Interaction** |
| **Effect per months extra use of plumpy'nut (Assuming 6 months use if not stopping after 1 month)** | **0.065**  **(-0.002 , 0.132)** | **0.022**  **(-0.047 , 0.091)** | **0.086**  **(0.017 , 0.155)** | **0.028**  **(-0.042 , 0.098)** |
| **Weeks (up to 16 weeks)** | 0.041  (0.033 , 0.048) | 0.012  (0.002 , 0.022) | 0.040  (0.032 , 0.048) | 0.013  (0.003 , 0.023) |
| **Weeks (additional after 16 weeks)** | 0.013  (0.010 , 0.016) | -0.0004  (-0.004 , 0.003) | 0.014  (0.011 , 0.017) | -0.001  (-0.005 , 0.002) |
| **Base WAZ** | 0.715  (0.644 , 0.787) | 0.990  (0.905 , 1.075) | 0.710  (0.636 , 0.784) | 0.984  (0.893 , 1.074) |
| **Base WAZ x weeks (up to 16 weeks)** |  | -0.015 (  -0.020 , -0.010) |  | -0.014  (-0.019 , -0.009) |
| **Base WAZ x weeks (additional after 16 weeks)** |  | -0.007  (-0.009 , -0.005) |  | -0.008  (-0.010 , -0.006) |
| **Age** | 0.050  (-0.121 , 0.221) | 0.0380  (-0.132 , 0.208) | 0.018  (-0.160 , 0.196) | 0.019  (-0.155 , 0.192) |
| **Age (additional spline term)** | -0.023  (-0.059 , 0.013) | -0.023  (-0.058 , 0.013) | -0.017  (-0.054 , 0.020) | -0.019  (-0.055 , 0.017) |
| **Primary carer (mother vs other)** | 0.037  (-0.131 , 0.204) | 0.035  (-0.133 , 0.204) | 0.064  (-0.094 , 0.222) | 0.061  (-0.095 , 0.216) |
| **Baseline WHO Stage (vs 1)** |  |  |  |  |
| 2 | 0.212  (-0.369 , 0.793) | 0.210  (-0.354 , 0.774) | 0.157  (-0.363 , 0.678) | 0.151  (-0.354 , 0.656) |
| 3 | 0.307  (-0.278 , 0.892) | 0.299  (-0.268 , 0.866) | 0.280  (-0.237 , 0.798) | 0.261  (-0.238 , 0.761) |
| 4 | 0.497  (-0.128 , 1.122) | 0.4896  (-0.117 , 1.096) | 0.403  (-0.142 , 0.949) | 0.397  (-0.131 , 0.925) |
| **Female vs Male** | -0.013  (-0.163 , 0.136) | -0.011  (-0.160 , 0.138) | -0.015  (-0.164 , 0.134) | -0.018  (-0.162 , 0.126) |
| **Baseline malnutrition indicator (Yes vs No)** | -0.035  (-0.248 , 0.178) | -0.011  (-0.223 , 0.201) | -0.013  (-0.229 , 0.204) | 0.030  (-0.187 , 0.247) |
| **Baseline CD4 (%) (per % increase)** |  |  | -0.008  (-0.016 , 0.000) |  |
| **Centre (vs Entebbe)** |  |  |  |  |
| JCRC | 0.019  (-0.220 , 0.259) | -0.021  (-0.260 , 0.217) | 0.056  (-0.183 , 0.295) | 0.008  (-0.220 , 0.235) |
| PIDC | 0.042  (-0.166 , 0.250) | -0.008  (-0.215 , 0.200) | 0.092  (-0.115 , 0.298) | 0.039  (-0.161 , 0.239) |
| **Constant** | -0.948  (-1.652 , -0.243) | -0.334  (-1.017 , 0.349) | -0.902  (-1.535 , -0.268) | -0.299  (-0.914 , 0.315) |

**Table S6: Full model output for primary analysis of effect of cumulative plumpy’nut use on weight for age in the first year after ART initiation in children with HIV aged less than 6 years from three study sites of the ARROW trial.**

|  | **Un-weighted** | | **IPTW** | |
| --- | --- | --- | --- | --- |
|  | **No interaction** | **Interaction** | **No interaction** | **Interaction** |
| **Effect per months extra use of plumpy'nut (Assuming 6 months use if not stopping after 1 month)** | **0.095**  **(-0.025 , 0.215)** | **0.030**  **(-0.091 , 0.151)** | **0.145**  **(0.021 , 0.268)** | **0.055**  **(-0.068 , 0.178)** |
| **Weeks (up to 16 weeks)** | 0.040  (0.033 , 0.048) | 0.012  (0.002 , 0.022) | 0.039  (0.031 , 0.047) | 0.013  (0.003 , 0.023) |
| **Weeks (additional after 16 weeks)** | 0.014  (0.011 , 0.016) | -0.0006  (-0.004 , 0.003) | 0.015  (0.011 , 0.018) | -0.002  (-0.005 , 0.002) |
| **Base WAZ** | 0.713  (0.641 , 0.785) | 0.991  (0.905 , 1.076) | 0.711  (0.638 , 0.783) | 0.979  (0.886 , 1.072) |
| **Base WAZ x weeks (up to 16 weeks)** |  | -0.015  (-0.020 , -0.010) |  | -0.014  (-0.019 , -0.008) |
| **Base WAZ x weeks (additional after 16 weeks)** |  | -0.007  (-0.009 , -0.005) |  | -0.008  (-0.010 , -0.006) |
| **Age** | 0.046  (-0.125 , 0.218) | 0.0365  (-0.134 , 0.207) | 0.019  (-0.158 , 0.196) | 0.019  (-0.154 , 0.193) |
| **Age (additional spline term)** | -0.023  (-0.059 , 0.013) | -0.022  (-0.058 , 0.013) | -0.017  (-0.054 , 0.019) | -0.019  (-0.055 , 0.017) |
| **Primary carer (mother vs other)** | 0.036  (-0.131 , 0.204) | 0.035  (-0.133 , 0.204) | 0.065  (-0.092 , 0.222) | 0.061  (-0.094 , 0.216) |
| **Baseline WHO Stage (vs 1)** |  |  |  |  |
| 2 | 0.210  (-0.367 , 0.788) | 0.209  (-0.353 , 0.772) | 0.153  (-0.366 , 0.672) | 0.150  (-0.355 , 0.655) |
| 3 | 0.304  (-0.277 , 0.885) | 0.297  (-0.268 , 0.862) | 0.275  (-0.241 , 0.791) | 0.261  (-0.239 , 0.761) |
| 4 | 0.491  (-0.130 , 1.112) | 0.4874  (-0.117 , 1.092) | 0.398  (-0.145 , 0.942) | 0.396  (-0.132 , 0.924) |
| **Female vs Male** | -0.014  (-0.164 , 0.135) | -0.011  (-0.160 , 0.138) | -0.018  (-0.165 , 0.130) | -0.019  (-0.163 , 0.125) |
| **Baseline malnutrition indicator (Yes vs No)** | -0.034  (-0.246 , 0.179) | -0.009  (-0.222 , 0.203) | -0.019  (-0.236 , 0.198) | 0.024  (-0.194 , 0.243) |
| **Baseline CD4 (%) (per % increase)** | -0.009  (-0.017 , 0.000) | -0.009  (-0.018 , 0.000) | -0.008  (-0.016 , 0.000) | -0.008  (-0.016 , 0.001) |
| **Centre (vs Entebbe)** |  |  |  |  |
| JCRC | 0.011  (-0.229 , 0.251) | -0.025  (-0.264 , 0.213) | 0.056  (-0.184 , 0.296) | 0.012  (-0.218 , 0.241) |
| PIDC | 0.030  (-0.180 , 0.240) | -0.013  (-0.222 , 0.196) | 0.088  (-0.122 , 0.297) | 0.043  (-0.160 , 0.246) |
| **Constant** | -0.934  (-1.635 , -0.233) | -0.325  (-1.007 , 0.356) | -0.886  (-1.518 , -0.255) | -0.308  (-0.925 , 0.310) |

**Table S7: Full model output for sensitivity analysis where duration of assumed use of plumpy’nut changed to 3 months.**

|  | **Un-weighted** | | **IPTW** | |
| --- | --- | --- | --- | --- |
|  | **No interaction** | **Interaction** | **No interaction** | **Interaction** |
| **Effect per months extra use of plumpy'nut (Assuming 6 months use if not stopping after 1 month)** | **0.052**  **(0.001 , 0.102)** | **0.016**  **(-0.038 , 0.069)** | **0.068**  **(0.014 , 0.122)** | **0.018**  **(-0.037 , 0.074)** |
| **Weeks (up to 16 weeks)** | 0.042  (0.034 , 0.049) | 0.012  (0.001 , 0.022) | 0.041  (0.034 , 0.049) | 0.012  (0.002 , 0.022) |
| **Weeks (additional after 16 weeks)** | 0.012  (0.009 , 0.015) | -0.0003  (-0.004 , 0.004) | 0.013  (0.010 , 0.017) | -0.001  (-0.005 , 0.003) |
| **Base WAZ** | 0.715  (0.644 , 0.786) | 0.993  (0.908 , 1.078) | 0.708  (0.633 , 0.782) | 0.987  (0.897 , 1.077) |
| **Base WAZ x weeks (up to 16 weeks)** |  | -0.015  (-0.020 , -0.010) |  | -0.015  (-0.019 , -0.010) |
| **Base WAZ x weeks (additional after 16 weeks)** |  | -0.007  (-0.009 , -0.004) |  | -0.008  (-0.010 , -0.006) |
| **Age** | 0.050  (-0.121 , 0.221) | 0.0373  (-0.133 , 0.208) | 0.017  (-0.162 , 0.196) | 0.018  (-0.156 , 0.192) |
| **Age (additional spline term)** | -0.023  (-0.060 , 0.013) | -0.023  (-0.058 , 0.013) | -0.017  (-0.055 , 0.020) | -0.019  (-0.056 , 0.017) |
| **Primary carer (mother vs other)** | 0.037  (-0.131 , 0.204) | 0.035  (-0.133 , 0.204) | 0.064  (-0.094 , 0.223) | 0.061  (-0.095 , 0.217) |
| **Baseline WHO Stage (vs 1)** |  |  |  |  |
| 2 | 0.213  (-0.368 , 0.795) | 0.210  (-0.354 , 0.773) | 0.159  (-0.363 , 0.680) | 0.151  (-0.353 , 0.654) |
| 3 | 0.308  (-0.278 , 0.894) | 0.298  (-0.268 , 0.865) | 0.282  (-0.236 , 0.800) | 0.260  (-0.238 , 0.759) |
| 4 | 0.498  (-0.128 , 1.124) | 0.4890  (-0.117 , 1.095) | 0.403  (-0.143 , 0.949) | 0.396  (-0.131 , 0.923) |
| **Female vs Male** | -0.014  (-0.163 , 0.136) | -0.011  (-0.160 , 0.138) | -0.015  (-0.164 , 0.135) | -0.018  (-0.163 , 0.126) |
| **Baseline malnutrition indicator (Yes vs No)** | -0.033  (-0.246 , 0.180) | -0.009  (-0.221 , 0.203) | -0.007  (-0.224 , 0.209) | 0.035  (-0.182 , 0.251) |
| **Baseline CD4 (%) (per % increase)** | -0.008  (-0.017 , 0.000) | -0.009  (-0.018 , 0.000) | -0.008  (-0.016 , 0.000) | -0.008  (-0.016 , 0.001) |
| **Centre (vs Entebbe)** |  |  |  |  |
| JCRC | 0.018  (-0.220 , 0.256) | -0.024  (-0.261 , 0.213) | 0.053  (-0.184 , 0.290) | 0.003  (-0.221 , 0.227) |
| PIDC | 0.040  (-0.166 , 0.247) | -0.011  (-0.216 , 0.195) | 0.087  (-0.116 , 0.291) | 0.034  (-0.163 , 0.231) |
| **Constant** | -0.955  (-1.660 , -0.251) | -0.328  (-1.011 , 0.355) | -0.909  (-1.542 , -0.275) | -0.290  (-0.902 , 0.322) |

**Table S8: Full model output for sensitivity analysis where duration of assumed use of plumpy’nut changed to 9 months.**


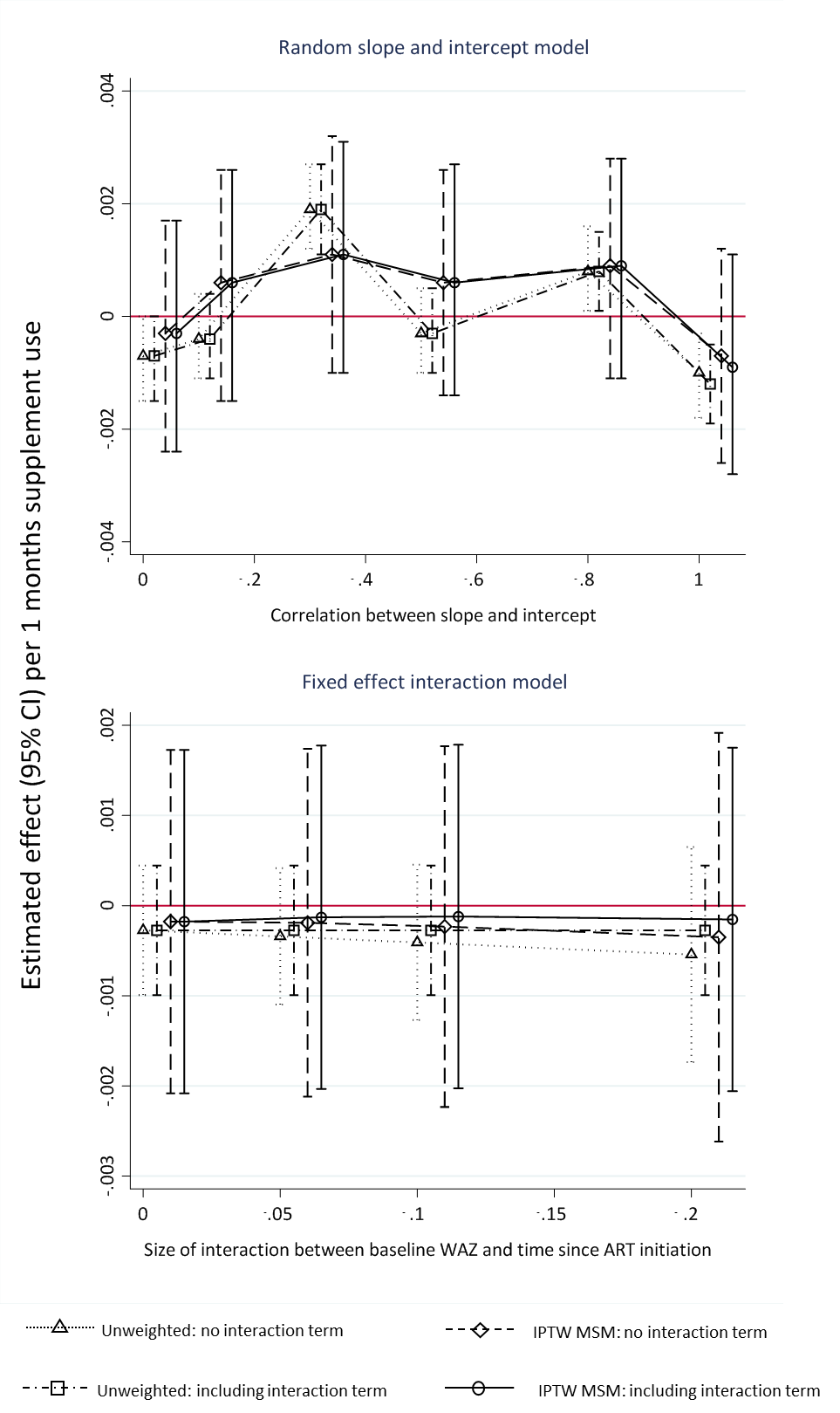


**Figure S1**: Effect (95% confidence intervals) of 1 month’s extra plumpy’nut on change in weight-for-age, where the true effect is zero, and plumpy’nut is allocated randomly at each time point in those yet to be treated; under different assumptions concerning the association between baseline weight-for-age and underlying weight-for-age trajectory. Association between baseline weight-for-age and slope simulated by a bivariate normal distribution (top) and a fixed effect interaction (bottom).
