# Supplementary material for: Marginal structural models for repeated measures where intercept and slope are correlated: An application exploring the benefit of nutritional supplements on weight gain in HIV-infected children initiating antiretroviral therapy
Source: PLoS One. 2020 Jul 9;15(7):e0233877. doi: 10.1371/journal.pone.0233877 (PMC7347189; doi:10.1371/journal.pone.0233877)
Supplement: S4 Appendix — (DOCX) [file pone.0233877.s004.docx]

/* Code for simulation where plumpy’nut allocation is NOT RANDOM, and intercept/slope correlation simulated using BIVARIATE NORMAL DISTRIBUTION */

*true effect of plumpy’nut varied through p

foreach p in 0 0.1 0.2 0.3 {

disp "effect = `p'"

drop _all

clear

set obs 2600000

set seed 85613456

set more off

*generate patid

gen sjid = _n

replace sjid = floor(_n/13)+1

replace sjid = 1 if sjid == 200001

bysort sjid: gen total_visits=_N

bysort sjid: gen visit = _n -1

sum total_visits

drop total_visits

*generate baseline weight for everyone

cap drop base_w

gen base_w = rnormal(-2,1.5) if visit==0

summ base_w

cap drop re1

gen re1 = base_w + 2

cap drop slope_*

bysort sjid: replace base_w = base_w[1]

*generate random slopes for varying correlation parameters

foreach s in 0 1 3 5 8 10 {

gen slope_`s' = -1*(`s'/10)*0.04*re1/1.5 + 0.04*sqrt(1-(-`s'/10)*(-`s'/10))*invnorm(uniform()) if visit==0

bysort sjid: replace slope_`s' = slope_`s'[1]

}

summ slope_* if base_w > 0

summ slope_* if base_w < 0 & base_w > -1

summ slope_* if base_w <-1 & base_w>-2

summ slope_* if base_w <-2 & base_w > -4

summ slope_* if base_w<-4

*generate trajectory

pause on

cap drop cur_w*

cap drop *plumpy*

cap drop pr_treat*

cap drop pr_stop*

foreach s in 0 1 3 5 8 10 {

gen cur_w_`s' = base_w if visit==0

gen plumpy_`s' = 0

gen cumu_plumpy_`s'=0

gen pr_treat_`s'=0

gen pr_stop_`s'=0

}

cap drop rand

gen rand = 1

foreach s in 0 1 3 5 8 10{

foreach v in 1 2 3 4 5 6 7 8 9 10 11 12{

*update cumulative plumpynut use only if no more than 4 months already

replace cumu_plumpy_`s' = cumu_plumpy_`s'[_n-1] + plumpy_`s'[_n-1] if visit==`v'

*update current weight based on previous weight, time and plumpynut use in previous month

replace cur_w_`s' = cur_w_`s'[_n-1] + (0.1 + slope_`s') + `p'*plumpy_`s'[_n-1] + rnormal(0,0.3) if visit==`v'

*generate probability of initiating treatment in those not yet treated

replace pr_treat_`s' = (exp(-1.5 - 1*cur_w_`s' - 0.1*base_w)/(1+exp(-1.5 - 1*cur_w_`s' - 0.1*base_w ))) if visit==`v' & cumu_plumpy_`s'==0

replace pr_treat_`s' = 1 if plumpy_`s'[_n-1]==1 & visit==`v'

*allocate treatment based on probability

replace rand = runiform(0,1) if visit==`v'

replace plumpy_`s' = 1 if rand < pr_treat_`s' & pr_treat_`s'!=. & visit==`v'

*in those on treatment, generate probability of stopping treatment

replace pr_stop_`s' = (exp(-1.38 + 0.5*cur_w_`s')/(1+exp(-1.38 + 0.5*cur_w_`s' ))) if visit==`v' & plumpy_`s'[_n-1]==1

*allocate stopping

replace rand = runiform(0,1) if visit==`v'

replace plumpy_`s' = 0 if rand < pr_stop_`s' & pr_stop_`s'!=. & visit==`v'

}

}

***** OUTCOME MODELS*********

set more off

xtset sjid

foreach s in 0 1 3 5 8 10 {

*unweighted models

xtgee cur_w_`s' cumu_plumpy_`s' visit if visit>0 , corr(ind)

est store unw_v_`s'

xtgee cur_w_`s' cumu_plumpy_`s' visit base_w if visit>0 , corr(ind)

est store unw_vbw_`s'

xtgee cur_w_`s' cumu_plumpy_`s' visit base_w c.visit#c.base_w if visit>0 , corr(ind)

est store unw_vbwint_`s'

****initiation weights***

*generate indicator for contributing to the initiation model

cap drop iptw_mod

gen iptw_mod = 1

replace iptw_mod = 0 if cumu_plumpy_`s'>0

*fit denominator model and calculate unstabilised weight

logistic plumpy_`s' cur_w_`s' base_w visit if visit>0 & iptw_mod==1

cap drop denom*

cap drop numer*

predict denom if e(sample)

replace denom = denom*(plumpy_`s') + (1-denom)*(1-plumpy_`s')

replace denom = 1 if iptw_mod==0

gen denom_w = denom if visit==1

replace denom_w = denom_w[_n-1]*denom if visit>1

cap drop usw

gen usw = 1/denom_w

summ usw, d

*fit numerator model and calculate stabilised weight

logistic plumpy_`s' visit base_w if visit>0 & iptw_mod==1

predict numer if e(sample)

replace numer = numer*(plumpy_`s') + (1-numer)*(1-plumpy_`s')

replace numer = 1 if iptw_mod==0

gen numer_w = numer if visit==1

replace numer_w = numer_w[_n-1]*numer if visit>1

cap drop iptw

gen iptw = numer_w/denom_w

cap drop iptw_lag

gen iptw_lag = iptw[_n-1] if sjid==sjid[_n-1]

replace iptw_lag=1 if visit==1

summ iptw_lag, d

***stopping weights***

*generate indicator for contributing to the stopping model

cap drop stop_mod

gen stop_mod = 0

replace stop_mod = 1 if cumu_plumpy_`s'>0

replace stop_mod=0 if cumu_plumpy_`s'>0 & plumpy_`s'[_n-1]==0 & sjid==sjid[_n-1]

*denominator model

logistic plumpy_`s' visit base_w cur_w_`s' cumu_plumpy_`s' if visit>1 & stop_mod==1

cap drop denom*

cap drop numer*

predict denom if e(sample)

replace denom = denom*(plumpy_`s') + (1-denom)*(1-plumpy_`s')

replace denom = 1 if stop_mod==0

gen denom_w = denom if visit==2

replace denom_w = denom_w[_n-1]*denom if visit>2

cap drop usw_stop

gen usw_stop = 1/denom_w

summ usw_stop, d

cap drop usw_stop_lag

gen usw_stop_lag = usw_stop[_n-1] if sjid==sjid[_n-1]

replace usw_stop_lag = 1 if visit<3

*numerator model

logistic plumpy_`s' visit base_w cumu_plumpy_`s' if visit>0 & stop_mod==1

predict numer if e(sample)

replace numer = numer*(plumpy_`s') + (1-numer)*(1-plumpy_`s')

replace numer = 1 if stop_mod==0

gen numer_w = numer if visit==2

replace numer_w = numer_w[_n-1]*numer if visit>2

*stabilised stopping weight

cap drop iptw_stop

gen iptw_stop = numer_w/denom_w

cap drop iptw_stop_lag

gen iptw_stop_lag = iptw_stop[_n-1] if sjid==sjid[_n-1]

replace iptw_stop_lag=1 if visit<3

summ iptw_stop_lag, d

*combine starting and stopping weights

cap drop weight

gen weight = iptw_lag*iptw_stop_lag

summ weight

*weighted outcome model

glm cur_w_`s' cumu_plumpy_`s' visit base_w [pw=weight] if visit>0, vce(cluster sjid)

est store w_noint_`s'

glm cur_w_`s' cumu_plumpy_`s' visit base_w c.visit#c.base_w [pw=weight] if visit>0, vce(cluster sjid)

est store w_int_`s'

estout unw_v_`s' unw_vbw_`s' unw_vbwint_`s' w_noint_`s' w_int_`s' using "sim_nonrandom_plumpy_randomslope.txt", append c("b(fmt(4)) ci_l(fmt(4)) ci_u(fmt(4))")

}

}

/* Code for simulation where plumpy’nut allocation is RANDOM, and intercept/slope correlation simulated using BIVARIATE NORMAL DISTRIBUTION*/

*true effect of plumpy’nut varied through p

foreach p in 0 0.1 0.2 0.3{

disp "effect = `p'"

drop _all

clear

set obs 2600000

set seed 234567

set more off

*generate patid

gen sjid = _n

replace sjid = floor(_n/13)+1

replace sjid = 1 if sjid == 200001

bysort sjid: gen total_visits=_N

bysort sjid: gen visit = _n -1

sum total_visits

drop total_visits

*generate baseline weight for everyone

cap drop base_w

gen base_w = rnormal(-2,1.5) if visit==0

summ base_w

cap drop re1

gen re1 = base_w + 2

cap drop slope_*

bysort sjid: replace base_w = base_w[1]

* generate each child’s set of random slopes

foreach s in 0 1 3 5 8 10 {

gen slope_`s' = -1*(`s'/10)*0.04*re1/1.5 + 0.04*sqrt(1-(-`s'/10)*(-`s'/10))*invnorm(uniform()) if visit==0

bysort sjid: replace slope_`s' = slope_`s'[1]

}

*check generated slopes

summ slope_* if base_w > 0

summ slope_* if base_w < 0 & base_w > -1

summ slope_* if base_w <-1 & base_w>-2

summ slope_* if base_w <-2 & base_w > -4

summ slope_* if base_w<-4

*generate weight for age trajectory for each correlation between slope and intercept

pause on

cap drop cur_w*

cap drop *plumpy*

cap drop pr_treat*

foreach s in 0 1 3 5 8 10 {

gen cur_w_`s' = base_w if visit==0

gen plumpy_`s' = 0

gen cumu_plumpy_`s'=0

gen pr_treat_`s'=0

}

cap drop rand

gen rand = 1

foreach s in 0 1 3 5 8 10 {

foreach v in 1 2 3 4 5 6 7 8 9 10 11 12 {

*update cumulative plumpynut use only if no more than 4 months already

replace cumu_plumpy_`s' = cumu_plumpy_`s'[_n-1] + plumpy_`s'[_n-1] if cumu_plumpy_`s'[_n-1]<=4 & visit==`v'

replace cumu_plumpy_`s' = cumu_plumpy_`s'[_n-1] if cumu_plumpy_`s'[_n-1]>4 & visit==`v'

*update current weight based on previous weight, time and plumpynut use in previous month

replace cur_w_`s' = cur_w_`s'[_n-1] + (0.1 + slope_`s') + `p'*plumpy_`s'[_n-1] + rnormal(0,0.3) if visit==`v'

cap drop cur_w_ind

gen cur_w_ind = 0

replace cur_w_ind = 1 if cur_w_`s'<0

*make plumpy use this month guaranteed if on it in previous month

replace plumpy_`s'=1 if plumpy_`s'[_n-1]==1 & visit==`v'

*but stop plumpy use if already had 4 months

replace plumpy_`s'=0 if cumu_plumpy_`s'>=4 & visit==`v'

*in those not yet started plumpy, randomly allocate whether they are prescribed plumpy at this visit but overwrite for those who have already had 4 months of plumpynut

replace rand = runiform(0,1) if visit==`v'

replace rand = 1 if cumu_plumpy_`s'>=4

replace plumpy_`s' = 1 if rand <=0.2 & visit==`v'

}

}

***** OUTCOME MODELS*****

set more off

xtset sjid

*unweighted models*

foreach s in 0 1 3 5 8 10 {

xtgee cur_w_`s' cumu_plumpy_`s' visit if visit>0 , corr(ind)

est store unw_v_`s'

xtgee cur_w_`s' cumu_plumpy_`s' visit base_w if visit>0 , corr(ind)

est store unw_vbw_`s'

xtgee cur_w_`s' cumu_plumpy_`s' visit base_w c.visit#c.base_w if visit>0 , corr(ind)

est store unw_vbwint_`s'

*generate some iptw weights (random initiation and fixed duration so initiation weights only)

cap drop iptw_mod

gen iptw_mod = 0

replace iptw_mod = 1 if cumu_plumpy_`s'==0

*denominator

logistic plumpy_`s' cur_w_`s' base_w visit if visit>0 & iptw_mod==1

cap drop denom*

cap drop numer*

predict denom if e(sample)

replace denom = denom*(plumpy_`s') + (1-denom)*(1-plumpy_`s')

replace denom = 1 if iptw_mod==0

gen denom_w = denom if visit==1

replace denom_w = denom_w[_n-1]*denom if visit>1

cap drop usw

gen usw = 1/denom_w

summ usw, d

*numerator

logistic plumpy_`s' visit base_w if visit>0 & iptw_mod==1

predict numer if e(sample)

replace numer = numer*(plumpy_`s') + (1-numer)*(1-plumpy_`s')

replace numer = 1 if iptw_mod==0

gen numer_w = numer if visit==1

replace numer_w = numer_w[_n-1]*numer if visit>1

*generate stabilised weight

cap drop iptw

gen iptw = numer_w/denom_w

cap drop iptw_lag

gen iptw_lag = iptw[_n-1] if sjid==sjid[_n-1]

replace iptw_lag=1 if visit==1

summ iptw_lag, d

*fit MSMs*

glm cur_w_`s' cumu_plumpy_`s' visit base_w [pw=iptw_lag] if visit>0, vce(cluster sjid)

est store w_noint_`s'

glm cur_w_`s' cumu_plumpy_`s' visit base_w c.visit#c.base_w [pw=iptw_lag] if visit>0, vce(cluster sjid)

est store w_int_`s'

estout unw_v_`s' unw_vbw_`s' unw_vbwint_`s' w_noint_`s' w_int_`s' using "sim_random_plumpy_randomslope.txt", append c("b(fmt(4)) ci_l(fmt(4)) ci_u(fmt(4))")

}

}

/* Code for simulation where plumpy’nut allocation is NOT RANDOM, and intercept/slope correlation simulated using FIXED EFFECT INTERACTION*/

*true effect of plumpy’nut varied through p

foreach p in 0 0.1 0.2 0.3{

disp "effect = `p'"

foreach x in 0 -0.05 -0.1 -0.2{

drop _all

clear

set obs 2600000

set seed 2

*generate patid

gen sjid = _n

replace sjid = floor(_n/13)+1

replace sjid = 1 if sjid == 200001

bysort sjid: gen total_visits=_N

bysort sjid: gen visit = _n -1

sum total_visits

drop total_visits

*generate baseline weight for everyone

cap drop base_w

gen base_w = rnormal(-2,1.5) if visit==0

summ base_w, d

replace base_w = r(p99) if base_w > r(p99)

replace base_w = r(p1) if base_w < r(p1)

summ base_w, d

bysort sjid: replace base_w = base_w[1]

*generate weight for age trajectory

*generate starting point for all, no one is on plumpy at baseline

cap drop cur_w*

cap drop *plumpy*

gen cur_w = base_w if visit==0

gen plumpy = 0

gen cumu_plumpy=0

cap drop pr_treat

gen pr_treat=0

cap drop rand

gen rand = 1

cap drop pr_stop

gen pr_stop=0

foreach v in 1 2 3 4 5 6 7 8 9 10 11 12 {

*update cumulative plumpynut

replace cumu_plumpy = cumu_plumpy[_n-1] + plumpy[_n-1] if visit==`v'

*update current weight

replace cur_w = cur_w[_n-1] + 0.1 + `p'*plumpy[_n-1] + rnormal(0,0.3) + `x'*base_w if visit==`v'

cap drop cur_w_ind

gen cur_w_ind = 0

replace cur_w_ind = 1 if cur_w<0

*generate probability of initiating treatment at this visit

replace pr_treat = (exp(-1.5 - 1*cur_w - 0.1*base_w)/(1+exp(-1.5 - 1*cur_w - 0.1*base_w ))) if visit==`v' & cumu_plump==0

replace pr_treat = 1 if plumpy[_n-1]==1 & visit==`v'

*allocate treatment based on probability

replace rand = runiform(0,1) if visit==`v'

replace plumpy = 1 if rand < pr_treat & pr_treat!=. & visit==`v'

*in those on treatment, generate probability of stopping treatment

replace pr_stop = (exp(-1.38 + 0.5*cur_w)/(1+exp(-1.38 + 0.5*cur_w ))) if visit==`v' & plumpy[_n-1]==1

*allocate stopping

replace rand = runiform(0,1) if visit==`v'

replace plumpy = 0 if rand < pr_stop & pr_stop!=. & visit==`v'

}

***** OUTCOME MODELS ********

*unweighted*

cap drop prev_w

gen prev_w = cur_w[_n-1] if sjid==sjid[_n-1]

gen plumpy_lag = 0 if visit==1

replace plumpy_lag=plumpy[_n-1] if sjid==sjid[_n-1] & visit>1

xtset sjid

disp "effect = `p'"

xtgee cur_w c.cumu_plumpy visit if visit>0 , corr(ind)

est store unw_v

xtgee cur_w c.cumu_plumpy visit base_w if visit>0 , corr(ind)

est store unw_vbw

xtgee cur_w c.cumu_plumpy visit base_w c.visit#c.base_w if visit>0 , corr(ind)

est store unw_vbwint

*generate some iptw weights by fitting separate start and stop models...

***initiation weights***

*generate indicator for contributing to the initiation model

cap drop iptw_mod

gen iptw_mod = 1

replace iptw_mod = 0 if cumu_plumpy>0

*fit denominator model and calculate unstabilised weight

logistic plumpy cur_w base_w visit if visit>0 & iptw_mod==1

cap drop denom*

cap drop numer*

predict denom if e(sample)

replace denom = denom*(plumpy) + (1-denom)*(1-plumpy)

replace denom = 1 if iptw_mod==0

gen denom_w = denom if visit==1

replace denom_w = denom_w[_n-1]*denom if visit>1

cap drop usw

gen usw = 1/denom_w

summ usw, d

cap drop usw_lag

gen usw_lag = usw[_n-1] if sjid==sjid[_n-1]

replace usw_lag = 1 if visit==1

*fit numerator model and calculate stabilised weight

logistic plumpy visit base_w if visit>0 & iptw_mod==1

predict numer if e(sample)

replace numer = numer*(plumpy) + (1-numer)*(1-plumpy)

replace numer = 1 if iptw_mod==0

gen numer_w = numer if visit==1

replace numer_w = numer_w[_n-1]*numer if visit>1

cap drop iptw

gen iptw = numer_w/denom_w

cap drop iptw_lag

gen iptw_lag = iptw[_n-1] if sjid==sjid[_n-1]

replace iptw_lag=1 if visit==1

summ iptw_lag, d

***stopping weights***

*generate indicator for contributing to the stopping model

cap drop stop_mod

gen stop_mod = 0

replace stop_mod = 1 if cumu_plumpy>0

replace stop_mod=0 if cumu_plumpy>0 & plumpy[_n-1]==0 & sjid==sjid[_n-1]

*denominator model

logistic plumpy visit base_w cur_w cumu_plumpy plumpy_lag if visit>1 & stop_mod==1

cap drop denom*

cap drop numer*

predict denom if e(sample)

replace denom = denom*(plumpy) + (1-denom)*(1-plumpy)

replace denom = 1 if stop_mod==0

gen denom_w = denom if visit==2

replace denom_w = denom_w[_n-1]*denom if visit>2

cap drop usw_stop

gen usw_stop = 1/denom_w

summ usw_stop, d

cap drop usw_stop_lag

gen usw_stop_lag = usw_stop[_n-1] if sjid==sjid[_n-1]

replace usw_stop_lag = 1 if visit<3

*numerator model

logistic plumpy visit base_w cumu_plumpy plumpy_lag if visit>0 & stop_mod==1

predict numer if e(sample)

replace numer = numer*(plumpy) + (1-numer)*(1-plumpy)

replace numer = 1 if stop_mod==0

gen numer_w = numer if visit==2

replace numer_w = numer_w[_n-1]*numer if visit>2

*stabilised stopping weight

cap drop iptw_stop

gen iptw_stop = numer_w/denom_w

cap drop iptw_stop_lag

gen iptw_stop_lag = iptw_stop[_n-1] if sjid==sjid[_n-1]

replace iptw_stop_lag=1 if visit<3

summ iptw_stop_lag, d

*combine starting and stopping weights

cap drop weight

gen weight = iptw_lag*iptw_stop_lag

summ weight

*fit MSMs

disp "effect = `p'"

disp "interaction = `x'"

glm cur_w c.cumu_plumpy visit base_w [pw=weight] if visit>0 , vce(cluster sjid)

est store w_noint

glm cur_w c.cumu_plumpy visit base_w c.visit#c.base_w [pw=weight] if visit>0, vce(cluster sjid)

est store w_int

estout unw_v unw_vbw unw_vbwint w_noint w_int using "sim_nonrandom_plumpy_timeweightinteraction.txt", append c("b(fmt(4)) ci_l(fmt(4)) ci_u(fmt(4))")

}

}

/* Code for simulation where plumpy’nut allocation is RANDOM, and intercept/slope correlation simulated using FIXED EFFECT INTERACTION*/

*true effect of plumpy’nut varied through p

foreach p in 0 0.1 0.2 0.3{

disp "effect = `p'"

foreach x in 0 -0.05 -0.1 -0.2{

drop _all

clear

set obs 2600000

set seed 12345678

*generate patid

gen sjid = _n

replace sjid = floor(_n/13)+1

replace sjid = 1 if sjid == 200001

bysort sjid: gen total_visits=_N

bysort sjid: gen visit = _n -1

sum total_visits

drop total_visits

*generate baseline weight for everyone

cap drop base_w

gen base_w = rnormal(-2,1.5) if visit==0

summ base_w, d

replace base_w = r(p99) if base_w > r(p99)

replace base_w = r(p1) if base_w < r(p1)

summ base_w, d

bysort sjid: replace base_w = base_w[1]

*generate weight for age trajectory

pause on

cap drop cur_w*

cap drop *plumpy*

gen cur_w = base_w if visit==0

gen plumpy = 0

gen cumu_plumpy=0

cap drop rand

gen rand = 1

foreach v in 1 2 3 4 5 6 7 8 9 10 11 12 {

*update cumulative use

replace cumu_plumpy = cumu_plumpy[_n-1] + plumpy[_n-1] if visit==`v'

replace cumu_plumpy = cumu_plumpy[_n-1] if cumu_plumpy[_n-1]>4 & visit==`v'

*update current weight based on past weight, time and whether given plumpynut at last visit (i.e. assume effect of plumpy is use in prior month)

replace cur_w = cur_w[_n-1] + 0.1 + `p'*plumpy[_n-1] + rnormal(0,0.3) + `x'*base_w if visit==`v'

cap drop cur_w_ind

gen cur_w_ind = 0

replace cur_w_ind = 1 if cur_w<0

*make plumpy use this month guaranteed if on it in previous month

replace plumpy=1 if plumpy[_n-1]==1 & visit==`v'

*but stop plumpy use if already had 4 months

replace plumpy=0 if cumu_plumpy>=4 & visit==`v'

*in those not yet started plumpy, randomly allocate whether they are prescribed plumpy at this visit m but overwrite for those who have already had 4 months of plumpy

replace rand = runiform(0,1) if visit==`v'

replace rand = 1 if cumu_plumpy>=4

replace plumpy = 1 if rand <=0.2 & visit==`v'

}

**** OUTCOME MODELS****

*unweighted*

xtset sjid

disp "effect = `p'"

xtgee cur_w c.cumu_plumpy visit if visit>0 , corr(ind)

est store unw_v

xtgee cur_w c.cumu_plumpy visit base_w if visit>0 , corr(ind)

est store unw_vbw

xtgee cur_w c.cumu_plumpy visit base_w c.visit#c.base_w if visit>0 , corr(ind)

est store unw_vbwint

*generate some iptw weights - nb fixed duration of use after starting so fit initiation only models

*generate indicator for contributing to the initiation model

cap drop iptw_mod

gen iptw_mod = 1

replace iptw_mod = 0 if cumu_plumpy>0

*denominator model

logistic plumpy cur_w base_w visit if visit>0 & iptw_mod==1

cap drop denom*

cap drop numer*

predict denom if e(sample)

replace denom = denom*(plumpy) + (1-denom)*(1-plumpy)

replace denom = 1 if iptw_mod==0

gen denom_w = denom if visit==1

replace denom_w = denom_w[_n-1]*denom if visit>1

cap drop usw

gen usw = 1/denom_w

summ usw, d

gen usw_lag = usw[_n-1] if sjid==sjid[_n-1]

replace usw = 1 if visit==1

*numerator model

logistic plumpy visit base_w if visit>0 & iptw_mod==1

predict numer if e(sample)

replace numer = numer*(plumpy) + (1-numer)*(1-plumpy)

replace numer = 1 if iptw_mod==0

gen numer_w = numer if visit==1

replace numer_w = numer_w[_n-1]*numer if visit>1

cap drop iptw

*generate stabilised weight

gen iptw = numer_w/denom_w

cap drop iptw_lag

gen iptw_lag = iptw[_n-1] if sjid==sjid[_n-1]

replace iptw_lag=1 if visit==1

summ iptw_lag, d

*fit MSMs

disp "effect = `p'"

glm cur_w c.cumu_plumpy visit base_w [pw=iptw_lag] if visit>0 , vce(cluster sjid)

est store w_noint

glm cur_w c.cumu_plump visit base_w c.visit#c.base_w [pw=iptw_lag] if visit>0, vce(cluster sjid)

est store w_int

estout unw_v unw_vbw unw_vbwint w_noint w_int using "sim_random_plumpy_timeweightinteraction.txt", append c("b(fmt(7)) ci_l(fmt(7)) ci_u(fmt(7))")

}

}
