# Supplementary material for: Marginal structural models for repeated measures where intercept and slope are correlated: An application exploring the benefit of nutritional supplements on weight gain in HIV-infected children initiating antiretroviral therapy
Source: PLoS One. 2020 Jul 9;15(7):e0233877. doi: 10.1371/journal.pone.0233877 (PMC7347189; doi:10.1371/journal.pone.0233877)
Supplement: S6 Appendix — (DOCX) [file pone.0233877.s006.docx]

**ARROW Trial Team**

**MRC/UVRI Uganda Research Unit on AIDS, Entebbe, Uganda:** P Munderi, P Nahirya-Ntege, R Katuramu, J Lutaakome, F Nankya, G Nabulime, I Sekamatte, J Kyarimpa, A Ruberantwari, R Sebukyu, G Tushabe, D Wangi, M Musinguzi, M Aber, L Matama, D Nakitto-Kesi, P Kaleebu, S Nassimbwa, W Senyonga.

**Joint Clinical Research Centre, Kampala, Uganda**: P Mugyenyi, V Musiime, R Keishanyu, VD Afayo, J Bwomezi, J Byaruhanga, P Erimu, C Karungi, H Kizito, WS Namala, J Namusanje, R Nandugwa, TK Najjuko, E Natukunda, M Ndigendawani, SO Nsiyona, R Kibenge, B Bainomuhwezi, D Sseremba, J Tezikyabbiri, CS Tumusiime, A Balaba, A Mugumya, F Nghania, D Mwebesa, M Mutumba, E Bagurukira, F Odongo, S Mubokyi, M Ssenyonga, M Kasango, E Lutalo, P Oronon, ED Williams, O Senfuma, L Mugarura, J Nkalubo, S Abunyang, O Denis, R Lwalanda, I Nankya, E Ndashimye, E Nabulime, D Mulima.

**University of Zimbabwe, Harare, Zimbabwe:** KJ Nathoo, MF Bwakura-Dangarembizi, F Mapinge, E Chidziva, T Mhute, T Vhembo, R Mandidewa, M Chipiti, R Dzapasi, C Katanda D Nyoni, GC Tinago, J Bhiri, S Mudzingwa, D Muchabaiwa, M Phiri, V Masore, CC Marozva, SJ Maturure, S Tsikirayi, L Munetsi, KM Rashirai, J Steamer, R Nhema, W Bikwa, B Tambawoga, E Mufuka, M Munjoma, K Mataruka, Y Zviuya **Zvitambo, Harare:** P Kurira, K Mutasa.

**Baylor College of Medicine Children’s Foundation Uganda, Mulago Hospital Uganda:** A Kekitiinwa, P Musoke, S Bakeera-Kitaka, R Namuddu, P Kasirye, A Babirye, J Asello, S Nakalanzi, NC Ssemambo, J Nakafeero, J Tikabibamu, G Musoba, J Ssanyu, M Kisekka.

**MRC Clinical Trials Unit at UCL, London, UK:** DM Gibb, MJ Thomason, AS Walker, AD Cook, AJ Szubert, B Naidoo-James, MJ Spyer, C Male, AJ Glabay, LK Kendall, J Crawley, AJ Prendergast.

**Independent ARROW Trial Monitors:** I Machingura, S Ssenyonjo.

**Trial Steering Committee:** I Weller (Chair), E Luyirika, H Lyall, E Malianga, C Mwansambo, M Nyathi, F Miiro, DM Gibb, A Kekitiinwa, P Mugyenyi, P Munderi, KJ Nathoo, AS Walker; Observers S Kinn, M McNeil, M Roberts, W Snowden.

**Data and Safety Monitoring Committee:** A Breckenridge (Chair), A Pozniak, C Hill, J Matenga, J Tumwine.

**Endpoint Review Committee (independent members):** G Tudor-Williams (Chair), H Barigye, HA Mujuru, G Ndeezi; Observers: S Bakeera-Kitaka, MF Bwakura-Dangarembizi, J Crawley, V Musiime, P Nahirya-Ntege, A Prendergast, M Spyer.
